# HIF2**α** inhibits glutaminase clustering in mitochondria to sustain growth of clear cell renal cell carcinoma

**DOI:** 10.1172/jci.insight.182711

**Published:** 2025-10-30

**Authors:** Wencao Zhao, Sara M. Demczyszyn, Nathan J. Coffey, Yanqing Jiang, Boyoung Kim, Schuyler Bowers, Caitlyn Bowman, Michael C. Noji, Cholsoon Jang, M. Celeste Simon, Zoltan Arany, Boa Kim

**Affiliations:** 1 Department of Medicine, Cardiovascular Institute, and Institute of Diabetes Obesity and Metabolism, and; 2 The Abramson Family Cancer Research Institute, Department of Cell and Developmental Biology, Perelman School of Medicine, University of Pennsylvania, Philadelphia, Pennsylvania, USA.; 3 McAllister Heart Institute, School of Medicine, University of North Carolina, Chapel Hill, North Carolina, USA.; 4 Biology Department, Williams College, Williamstown, Massachusetts, USA.; 5 Department of Biological Chemistry, School of Medicine, University of California, Irvine, California, USA.; 6 Department of Pathology and Laboratory Medicine, McAllister Heart Institute, Nutrition Obesity Research Center, and Lineberger Cancer Center, University of North Carolina, Chapel Hill, North Carolina, USA.

**Keywords:** Cell biology, Metabolism, Cancer, Mitochondria

## Abstract

Clear cell renal cell carcinomas (ccRCCs) are largely driven by HIF2α and are avid consumers of glutamine. However, inhibitors of glutaminase 1 (GLS1), the first step in glutaminolysis, have not shown benefit in phase III trials, and HIF2α inhibition, recently FDA approved for treatment of ccRCC, shows significant but incomplete benefits. This highlights the need to better understand the interplay between glutamine metabolism and HIF2α in ccRCC. Here, we report that glutamine deprivation rapidly redistributed GLS1 into isolated clusters within mitochondria in diverse cell types, but not in ccRCC. GLS1 clustering occurred rapidly within 1–3 hours, was reversible, was specifically triggered by reduced intracellular glutamate, and was dependent on mitochondrial fission. Clustered GLS1 markedly enhanced glutaminase activity and promoted cell death under glutamine-deprived conditions. HIF2α prevented GLS1 clustering, independently of its transcriptional activity, thereby maintaining low GLS activity and protecting ccRCC cells from glutamine-deprivation-induced cell death. Forced clustering of GLS1, using constitutively clustering mutants, restored high GLS activity, promoted apoptosis, and suppressed ccRCC tumor growth in vivo. These findings reveal multiple insights into cellular glutamine handling, including a previously unrecognized process by which HIF2α promotes ccRCC: by suppressing GLS1 clustering and maintaining low GLS activity. This mechanism provides a potential explanation for the lack of clinical efficacy of GLS inhibitors in ccRCC and suggests a therapeutic avenue to combine HIF2α inhibition with strategies that restore GLS1 clustering.

## Abstract 2

The cancer-driving protein HIF2α blocks clustering and activation of glutaminase, allowing kidney tumor cells to keep growing and revealing a new therapeutic target.

## Graphical abstract

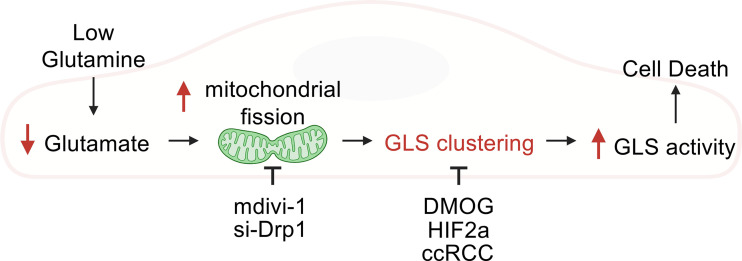

## Introduction

Glutamine is the most abundant amino acid in human blood and is crucial for cellular growth and survival (1–3). Glutamine is an important anaplerotic source of carbons for the tricarboxylic acid (TCA) cycle and a nitrogen source for various processes, thus contributing significantly to both ATP production and biomass synthesis in numerous cell types (2, 4–6). Glutamine is also essential for the synthesis of non-essential amino acids (NEAAs) such as asparagine, provides the backbone for glutathione in most cells, and is an important gluconeogenic precursor and regulator of urinary pH in the kidney (6). Depletion of glutamine induces cell death across diverse cell types, variably attributable to energy depletion, inhibition of the mTOR pathway, or the initiation of endoplasmic reticulum (ER) stress (4). The tumor microenvironment is often nutrient poor due to reduced vascularization and/or the hyperactive metabolism of cancer cells. Glutamine has been demonstrated by many studies (7–11), though not all (12, 13), to be one of the most depleted metabolites in tumors compared with corresponding normal tissues. Consistent with this fact, tumors are generally avid consumers of glutamine, thus creating a potential liability.

Glutaminase (GLS) mediates the initial step in glutamine catabolism, the conversion of glutamine to glutamate, releasing a single ammonium ion (14). There are 2 isozymes of GLS, GLS1 and -2, encoded by separate genes (15, 16). GLS2 expression is largely restricted to periportal hepatocytes, where the enzymes couple ammonia liberation to the production of urea (17). GLS1 is expressed in most tissues and cancer, and encodes 2 alternatively spliced isoforms, kidney glutaminase A (KGA), expressed largely in kidney, and the widely expressed and more active glutaminase isoform C (GAC) (16). All GLS1 enzymes are localized in the mitochondrial matrix (16). GLS1 is generally thought to be regulated by tetramerization from an inactive dimer, a process requiring inorganic phosphate (18, 19). More recent work has shown that GLS1 can further oligomerize into large filamentous structures with additional enhancement of enzymatic activity (20–23). The biological relevance of these findings is poorly understood.

Clear cell renal carcinoma (ccRCC) accounts for approximately 80% of renal malignancies (24), with a 5-year survival rate of approximately 50%, but reduced to approximately 10% when metastatic (25). ccRCC is usually driven by the genetic or epigenetic loss of von Hippel-Lindau tumor suppressor protein (pVHL, encoded by *VHL*) function (24). In familial cases of ccRCC, there is heterozygous inheritance of *VHL* mutations, with loss of heterozygosity in the tumors (26). Like many tumors, ccRCC tumors are avid consumers of glutamine, and GLS has thus long been entertained as a possible therapy for ccRCC. However, despite some efficiency with GLS inhibition in preclinical models, GLS inhibition was not effective in a recent phase III placebo-controlled, double-blind, randomized clinical trial (PCDB-RCT) (27). pVHL is a component of an E3 ubiquitin ligase complex required for the degradation of HIFs in the presence of oxygen. Loss of *VHL* in ccRCC is associated with stabilization of HIF, and HIF2α is likely the most important driver of ccRCC. Inhibition of HIF2α with belzufitan, a PT2385 analog, was approved for treatment of familial ccRCC in 2021, after a phase II, open-label study showed activity in patients with ccRCC, representing a first-in-class drug approval (28). The approval was expanded to all ccRCC in 2023 after a phase III PCDB-RCT showed marked improvements over everolimus (29). However, although impressive, responses in both studies were largely partial, and seen only in approximately 50% of patients (30). There is thus an urgent need to better understand both the handling of glutamine and the mechanisms of HIF2α action in ccRCC.

In this study, we investigated the molecular and cellular mechanisms of GLS1 regulation and their impact in ccRCC biology. While studying the effects of glutamine deprivation on endothelial cells, we noted dramatic clustering of GLS1 to discrete puncta throughout the cells. We used a range of pharmacological and genetic approaches to examine this process in depth, including its kinetics, its mechanism, and its impact on cellular GLS1 activity. Moreover, we identified HIF2α as a key regulator of GLS1 clustering, and demonstrate that constitutive GLS1 clustering suppresses ccRCC tumor grown in vivo.

## Results

### Glutamine deprivation uniquely triggers the clustering of GLS within mitochondria.

**Figure 1:**
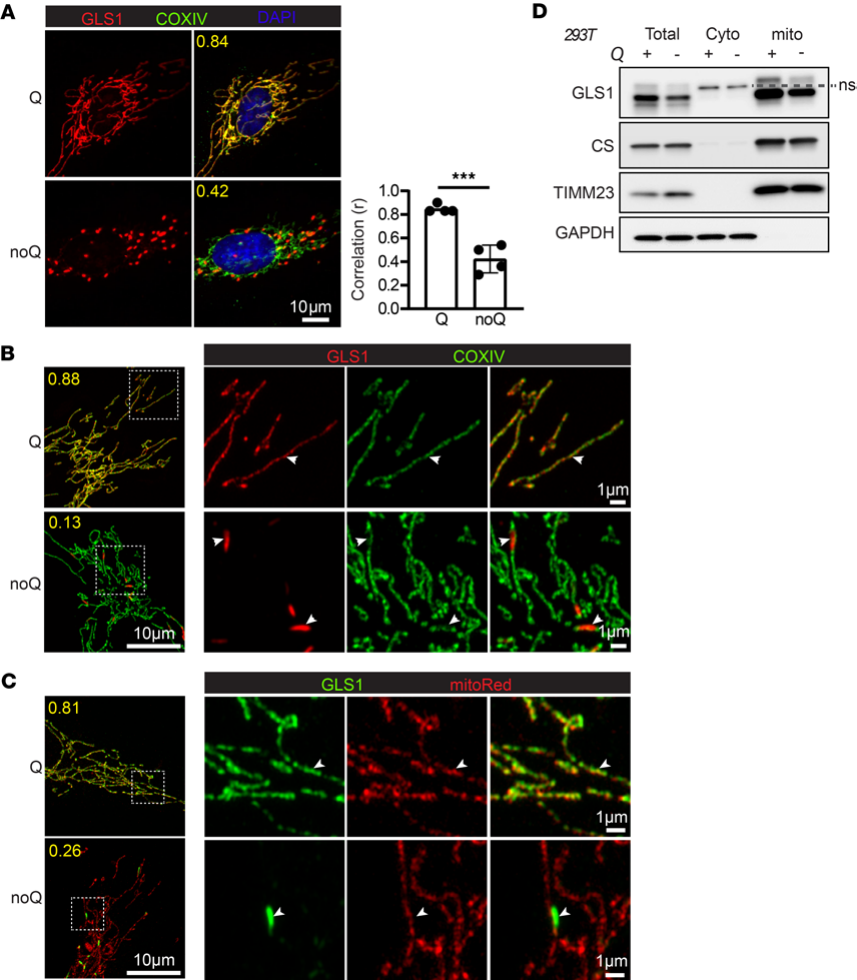
Glutamine deprivation induces GLS1 clustering within mitochondria. (**A**) Immunocytochemistry (ICC) of GLS1 (red) in HUVECs after 24-hour culture in glutamine-supplemented (Q) versus -deprived (noQ) media. GLS was costained for COXIV (green) and with DAPI (blue). Images were acquired with a wide-field fluorescence microscope using a 100× objective lens. Scale bar: 10 μm. The correlation coefficient (*r*) of GLS1 and COXIV staining was calculated using CellProfiler. ***P < 0.001 by 2-tailed Student’s *t* test. (**B**) ICC of GLS1 (red) and COXIV (green) after a 24-hour culture in Q versus noQ media followed by imaging using a confocal and Airyscan microscope. (**C**) Costaining of GLS1 (green) with MitoTracker Red dye after 24-hour culture in Q versus noQ media followed by imaging using a confocal and Airyscan microscope. Scale bars (**B** and **C**): 10 μm (left) and 1 μm (right). (**D**) Mitochondrial fractionation assay performed in 293T cells after 24-hour culture in Q (+) versus noQ (–) media.

While studying the effects of glutamine deprivation (noQ) on human umbilical vein endothelial cells (HUVECs) (31), we incidentally noted a striking redistribution of GLS1 to discrete puncta throughout the cell, as seen by immunocytochemistry (ICC) and wide-field fluorescence microscopy imaging, 24 hours after noQ (Figure 1A). Costaining of GLS1 with markers for various intracellular organelles, including Lamp1 for lysosomes, calnexin for endoplasmic reticulum, BODIPY dye for lipid droplets, and golgin for the Golgi apparatus, during noQ revealed no colocalization of GLS1 puncta with these organelles (Supplemental Figure 1). In contrast, confocal and Airyscan imaging and costaining for cytochrome *c* oxidase IV (COXIV) (Figure 1B) or MitoTracker Red (Figure 1C), both markers of mitochondria, demonstrated the noQ induced GLS1 puncta to represent clustering of GLS1 within mitochondria themselves. Biochemical cellular fractionation assays, coupled with Western blot analysis, confirmed the persistent presence of GLS1 within mitochondria following noQ treatment (Figure 1D). The clustering of GLS1 represented redistribution of existing GLS1 pools, rather than de novo–synthesized GLS1, because treating cells with the protein synthesis inhibitor cycloheximide prior to removing glutamine did not prevent clustering (Supplemental Figure 2). The phenomenon was also not restricted to HUVECs and was observed in other cell lines tested, including HeLa, 293T, HCT116, and HepG2 (Supplemental Figure 3). Finally, and importantly, noQ-induced clustering of GLS1 was unique, as it did not occur with other mitochondrial proteins, including glutamate dehydrogenase (GLUD), COXIV, citrate synthase (CS), pyruvate dehydrogenase (PDH), HADHA, and COX4-I1 (Supplemental Figure 4).

### GLS clustering is rapid and occurs within the physiological range of glutamine concentrations.

**Figure 2:**
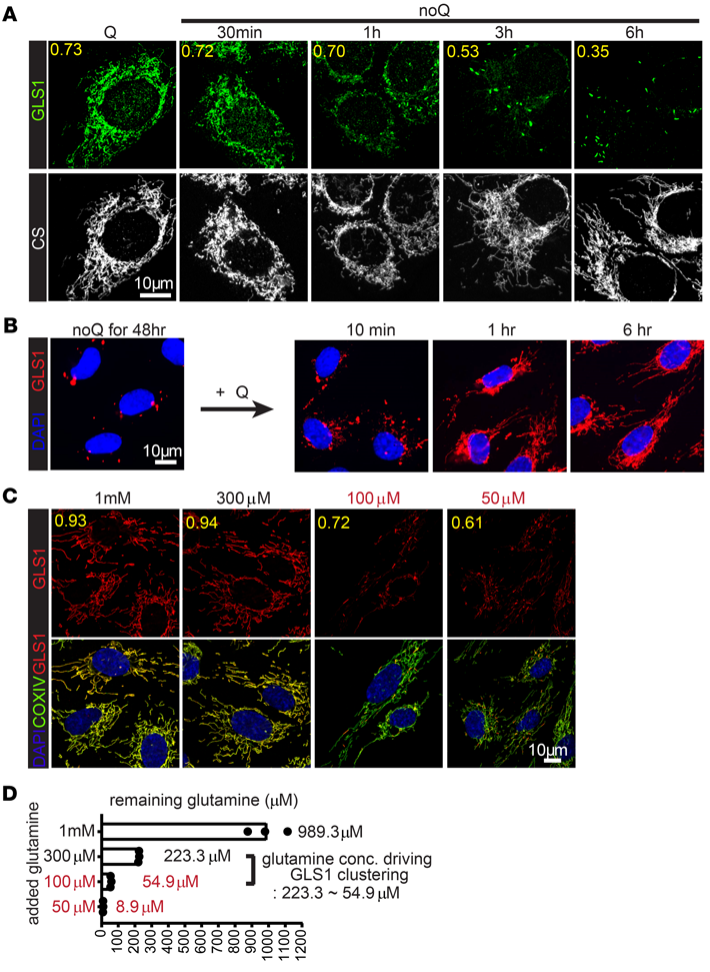
Time course and dose response of glutamine deprivation for GLS1 clustering. (**A**) noQ time-course study in C2C12 myoblasts. Costaining of GLS1 (green) with CS (gray) after noQ for the indicated time points. (**B**) Glutamine (2 mM) replenishment time-course study in HUVECs after 48 hours of noQ. GLS1 (red) and DAPI. (**C**) Glutamine dose-response study in HUVECs. Costaining of GLS1 (red) with COXIV (green) after incubation with the indicated concentrations of glutamine for 6 hours. GLS1 clustering was observed under conditions where 100 μM or 50 μM glutamine was used. (**D**) Concentration of the remaining glutamine (μM) measured in the media of the experimental conditions in **C**. Scale bars: 10 μm.

To determine the kinetics of GLS1 clustering, we carried out a time-course study in C2C12 cells. GLS1 clustering was observed within 1 hour of glutamine deprivation and was complete by 6 hours (Figure 2A). GLS1 clustering was also reversible, with similar kinetics, as replenishing glutamine led to the redistribution of clustered GLS1 as early as 1 hour (Figure 2B). To determine the concentration of glutamine below which GLS1 clustering occurs, we performed a dose-response study in HUVECs. Treating cells with 100 μM glutamine for 6 hours promoted GLS1 clustering, while 300 μM did not (Figure 2C). Because these cells are avid consumers of glutamine (31), we measured glutamine concentration in the media at the end of the 5-hour incubation, revealing a remaining 55 μM and 223 μM, respectively (Figure 2D), indicating that GLS1 clustering occurs within this range. Normal plasma concentrations of glutamine are approximately 500 μM, but intratumor or for example brain interstitial concentrations are much lower, the latter approximately 80 μM (1). GLS1 clustering thus occurs in a range of glutamine concentration that can be found in tumors and other nutrient-poor settings.

### GLS clustering is triggered by sensing glutamate levels.

**Figure 3:**
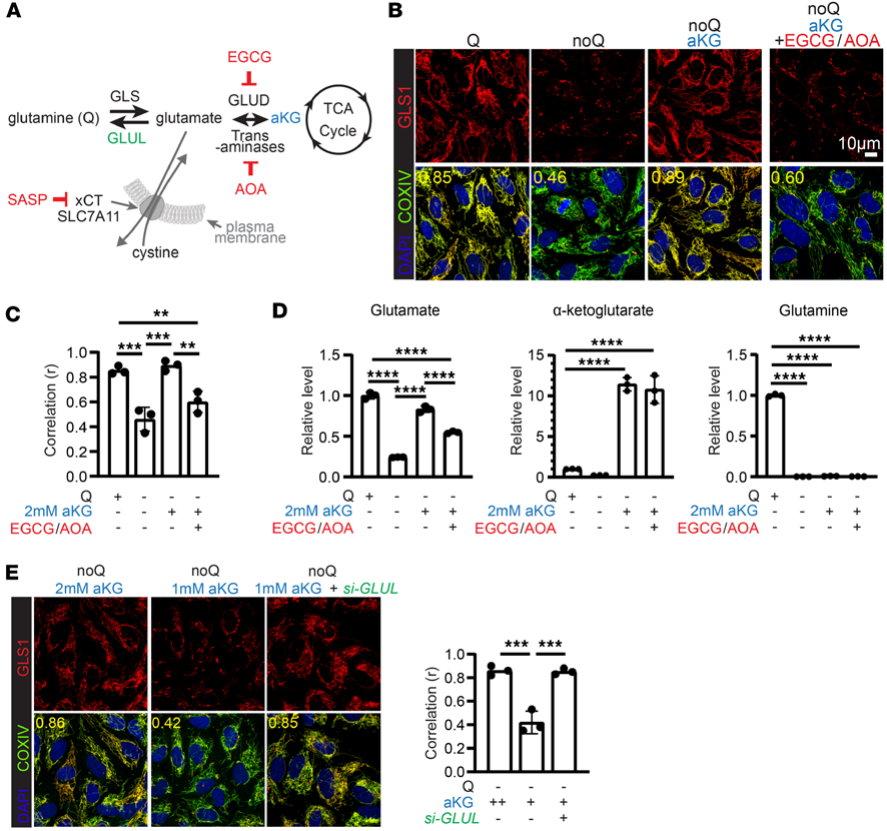
The level of glutamate, not glutamine, determines GLS1 clustering. (**A**) Schematic of the rescue study, where targeted pathways and proteins are highlighted. (**B**) Left: Rescue of GLS1 clustering by dimethyl-αKG (2 mM) supplementation in noQ for 6 hours. Right: Reversal of the dimethyl-αKG–mediated rescue of GLS clustering by 6-hour treatment with EGCG (100 μM) and AOA (500 μM). (**C**) Correlation coefficient (*r*) for the conditions in **B**. (**D**) Quantifications of cellular glutamate, αKG, and glutamine in HUVECs with conditions in **B**. (**E**) siRNA-mediated knockdown of GLUL promotes the rescue by dimethyl-αKG (1 mM) supplementation in noQ. **P < 0.01; ***P < 0.001; ****P < 0.0001 by 1-way ANOVA followed by Bonferroni’s post hoc testing. Scale bars: 10 μm.

We next investigated the mechanism by which GLS1 clustering is induced. We first sought to determine how the low-glutamine state is sensed. Upon cellular uptake, glutamine undergoes catabolism to glutamate by GLS1, followed by conversion to α-ketoglutarate (αKG) through the action of GLUD and transaminases. Subsequently, αKG serves as an anaplerotic carbon source entering the TCA cycle. Conversely, αKG can contribute to the de novo synthesis of glutamate and glutamine via GLUD, transaminases, and glutamine synthase (GLUL), as illustrated in the schematic diagram (Figure 3A). Supplementation with 2 mM dimethyl-αKG, a cell-permeable form of αKG, completely inhibited GLS1 clustering (Figure 3, B and C). Unmethylated αKG also prevented GLS1 clustering but required a higher concentration, consistent with poor cellular uptake (Supplemental Figure 5A). We have shown previously that glutamine deprivation profoundly reduces intracellular levels of TCA intermediates, and that dimethyl-αKG largely rescues this effect (31). Therefore, to dissect whether the rescue of GLS1 clustering by αKG relies on de novo synthesis of glutamate and glutamine versus its replenishment of TCA intermediates, we blocked αKG-to-glutamate conversion using EGCG and AOA, inhibitors of GLUD and transaminases, respectively (Figure 3, B and C). This treatment completely reversed the rescue of GLS1 clustering by αKG in noQ conditions, indicating that αKG-mediated rescue of GLS1 clustering is likely mediated by glutamate or glutamine, rather than replenishing TCA intermediates. Treatment with either EGCG or AOA did not fully reverse the αKG-mediated rescue of GLS1 clustering, consistent with redundancy in these pathways (Supplemental Figure 5B). αKG supplementation restored intracellular glutamate levels in noQ conditions, but did not restore glutamine levels (Figure 3D), suggesting that glutamate, rather than glutamine, likely regulates GLS1 clustering. To confirm this conclusion, we knocked down GLUL to inhibit glutamine synthesis from glutamate, which led to further enhancement of the αKG-mediated rescue of GLS1 clustering (Figure 3E), demonstrating that glutamate regulates GLS1 clustering independently of glutamine. Also consistent with this conclusion, supplementation with glutamate or monosodium glutamate (MSG) rescued GLS1 clustering (Supplemental Figure 5C), as did pharmacologically or genetically blocking the xCT antiporter, responsible for exporting glutamate out of the cell (Supplemental Figure 5D). Finally, considering the role of glutamine as a nitrogen source, we also tested whether nitrogen depletion contributes to GLS1 clustering, but supplementing cells in noQ with ammonia showed no effect on GLS1 clustering (Supplemental Figure 5E). We conclude that GLS1 clustering is triggered specifically by sensing glutamate.

### Mitochondrial fusion/fission is required for GLS clustering.

**Figure 4:**
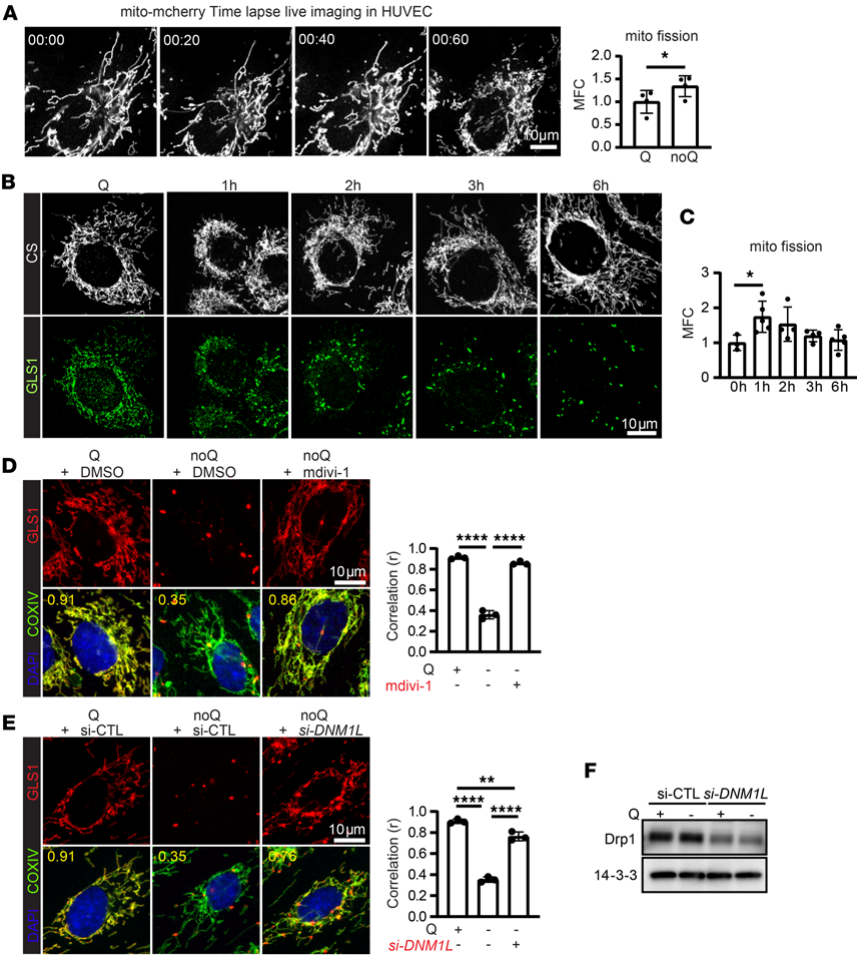
Mitochondrial fission is required for GLS1 clustering. (**A**) Time-lapse live imaging of mito-mCherry–overexpressing HUVECs during noQ. Quantification of mitochondrial fission is presented as mitochondrial fragmentation count (MFC). (**B** and **C**) ICC of CS and GLS1 during glutamine deprivation in C2C12 myoblasts. Quantification of mitochondrial fission is presented as MFC. (**D**) Rescue of GLS1 clustering by treatment with 20 μM mdivi-1. (**E**) Rescue of GLS clustering by siRNA knockdown of *DNM1L*. (**F**) Western blotting analysis of siRNA of DRP1 in HUVECs. *P < 0.05; **P < 0.01; ****P < 0.0001 by 2-tailed Student’s *t* test (**A** and **C**) or 1-way ANOVA followed by Bonferroni’s post hoc testing (**D** and **E**). Scale bars: 10 μm.

We next sought to determine by what process the redistribution of GLS1 within the large mitochondrial network occurs. Mitochondria are highly dynamic organelles that continually undergo fusion and fission processes. The equilibrium between fusion and fission, along with significant rearrangements in the mitochondrial network, is known to vary under different metabolic states. For example, prolonged nutrient deprivation, including glutamine deprivation, promotes mitochondrial elongation (32, 33). Strikingly, however, we observed that in the short term, glutamine deprivation caused a rapid and transient fragmentation of mitochondria; mitochondrial fission was observed within 1 hour of glutamine deprivation (Figure 4A), and the tubular-elongated mitochondrial network was restored within the subsequent hours, despite persistent deprivation of glutamine. Interestingly, the clustering of GLS1 in response to glutamine deprivation coincided with this transient mitochondrial fragmentation (Figure 4, B and C), suggesting that a fission/fusion cycle is required for the redistribution and microlocalization of GLS1 into puncta. To test this notion, we inhibited DRP1, a key effector of mitochondrial fission, either pharmacologically (mdivi-1) (Figure 4D) or by genetic knockdown of *DNM1L* (gene symbol for DRP1) (Figure 4, E and F). In both cases, inhibition of mitochondrial fission largely prevented GLS1 clustering. We conclude that mitochondrial fission is a requisite process for GLS1 clustering in response to glutamine deprivation, likely enabling the redistribution and segregation of GLS1 within the mitochondrial network.

### GLS clustering increases its enzymatic activity.

**Figure 5:**
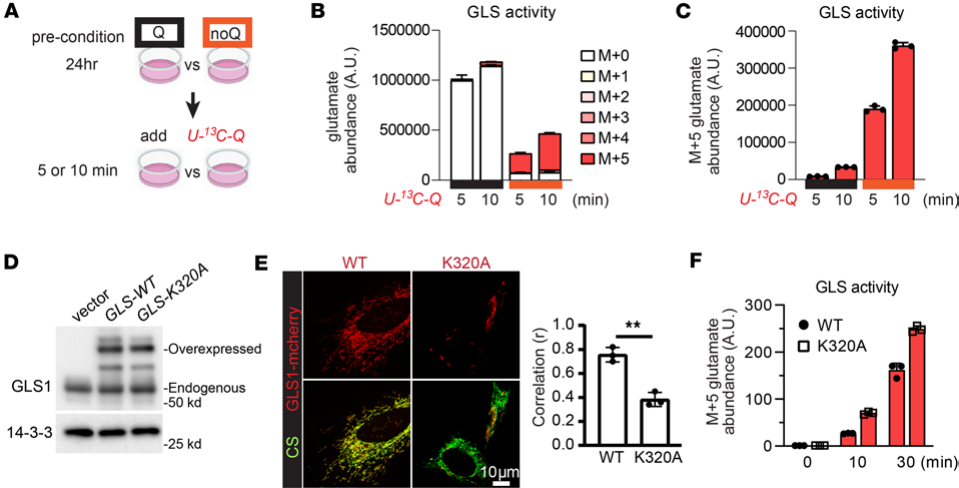
GLS1 clustering increases its enzymatic activity. (**A**) Schematic of the tracing experiment using [U-^13^C_5_]glutamine (Q) to measure enzymatic activity of GLS. (**B**) All glutamate that are heavy-isotope labeled by the indicated number of carbons. M+*n*: glutamate with *n* carbon atoms labeled with ^13^C. (**C**) Separate plot of ^13^C-glutamate that are M+5. (**D**) Western blotting analysis for the validation of the equivalent expression of GLS1-WT or GLS1-K320A in HeLa cells. Note: The constructs of GLS-WT and GLS-K320A were fused with mCherry and thus had a higher molecular weight than that of endogenous GLS. (**E**) ICC of WT versus K320A-GLS1-mCherry–overexpressing HeLa cells. Scale bar: 10 μm. **P < 0.01 by 2-tailed Student’s *t* test. (**F**) ^13^C-glutamate that are M+5 in WT versus K320A-GLS1-mCherry–overexpressing HeLa cells.

Prior biochemical studies have shown that GLS1, under some conditions such as high concentrations of inorganic phosphate, can form supratetrameric filamentous oligomers, and that this oligomerization boosted GLS1 enzymatic activity (19, 22). We therefore hypothesized that GLS1 clustering in response to glutamine deprivation may similarly boost enzymatic activity, perhaps as a physiological adaptation to low substrate availability. To test this notion in intact cells, we employed a heavy isotope–tracing approach. Cells were preconditioned for 24 hours in either glutamine-containing (Q) or -deprived (noQ) media to induce GLS1 clustering, and subsequently additionally exposed to [U-^13^C_5_]glutamine for 5 or 10 minutes, followed by quantification by mass spectrometry of heavy isotope–labeled glutamate, the product of the GLS1 reaction (schematic in Figure 5A). As shown in Figure 5, B and C, after 24 hours of glutamine deprivation, and thus in the context of GLS1 clustering, the amount of ^13^C-labeled glutamate (M+5) produced in 5 and 10 minutes was more than 10 times that seen in cells maintained in complete media. Clustering in response to glutamine deprivation thus dramatically increases GLS1 enzymatic activity. Previous reports indicating that GLS1 can form supratetrameric oligomers with enhanced enzymatic activity identified a critical lysine at position 320 (K320), mutation of which led to constitutive oligomerization (19, 22). Consistent with this, we found that expression of this K320A GLS1 mutant (Figure 5D) in intact cells leads to constitutive clustering of GLS1, even under glutamine-rich conditions (Figure 5E), and concomitant markedly higher GLS1 activity than in cells expressing wild-type (WT) GLS1, as determined by [U-^13^C_5_]glutamine tracing (Figure 5F). In sum, intracellular GLS1 clustering induced either by glutamine deprivation or by genetic manipulation markedly increases its enzymatic activity.

### HIF2α suppresses GLS clustering.

**Figure 6:**
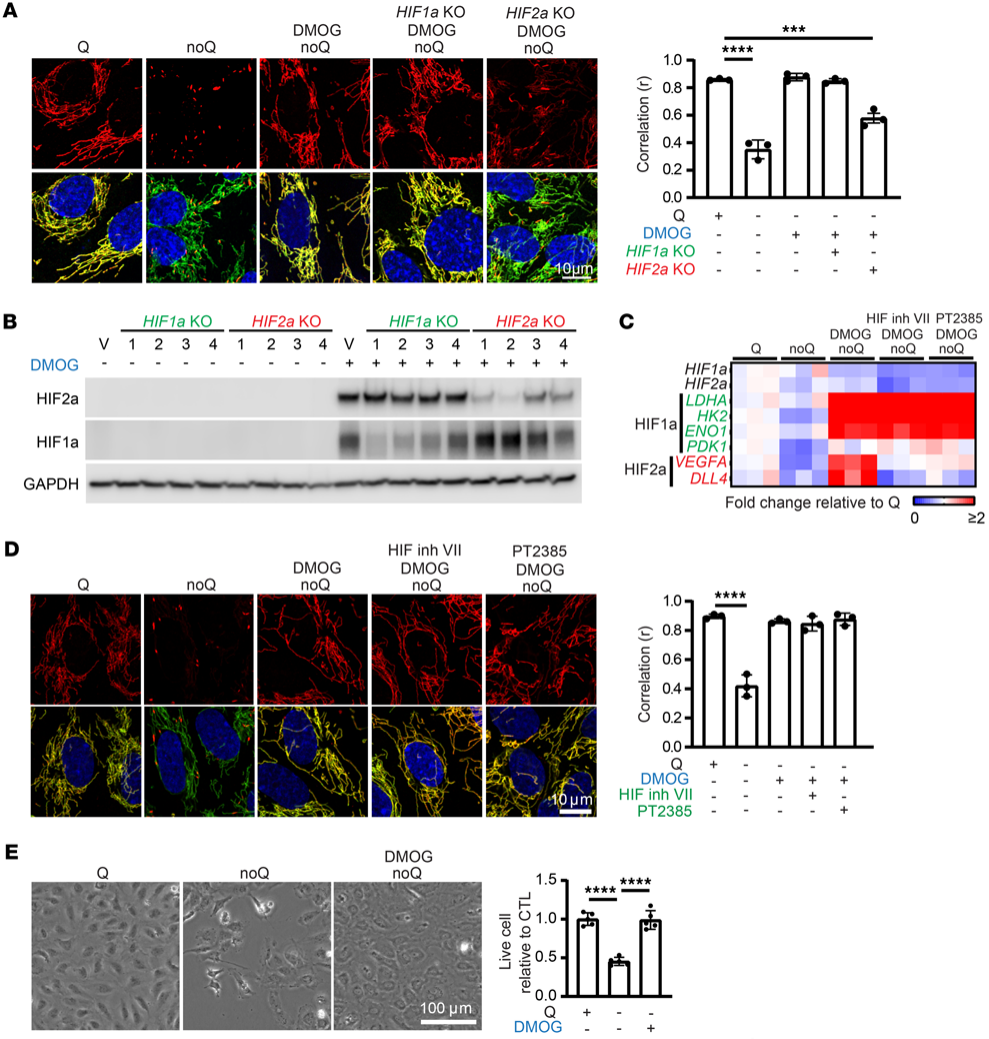
HIF2α prevents DMOG-induced redistribution of GLS1. (**A**) Rescue of GLS1 clustering by DMOG treatment is reversed by *HIF2α* but not *HIF1α* knockout (KO) in HUVECs. Correlation coefficient (*r*) for GLS1 (red) and COX IV (green) is shown on the right. (**B**) Western blotting analysis showing the validation of *HIF1α* KO and *HIF2α* KO in HUVECs. gRNAs are numbered 1–4 on top. V, vector. *HIF1α* gRNA1 and *HIF2α* gRNA2 were selected in the assays. (**C**) qPCR analysis showing the validation of DMOG and HIF2α transcriptional inhibitors in HUVECs. HIF1α target genes: *LDHA*, *HK2*, *ENO1*, and *PDK1*. HIF2α target genes: *VEGFA* and *DLL4*. (**D**) No reversal of the DMOG-induced GLS1 redistribution by inhibitors targeting the transcriptional activity of HIF2α. (**E**) Rescue of noQ-induced cell death by DMOG treatment in HUVECs. ***P < 0.001; ****P < 0.0001 by 1-way ANOVA followed by Bonferroni’s post hoc testing. Scale bars: 10 μm (**A** and **D**) and 100 μm (**E**).

In further efforts to elucidate the molecular mechanisms that drive GLS1 clustering, we tested the impact of several compounds that mimic aspects of tumor biology and identified the hypoxia-mimetic dimethyloxalylglycine (DMOG) as a potent suppressor of GLS1 clustering (Figure 6A and Supplemental Figure 6, A–D). DMOG is an αKG analog that inhibits several αKG-dependent dioxygenases, including HIF prolyl hydroxylase, which mediate degradation of HIF transcription factors in the presence of oxygen (34). Consistent with this, DMOG treatment stabilized HIF1α and HIF2α proteins (Figure 6B and Supplemental Figure 6E) and induced their transcriptional activity (Figure 6C). We hypothesized that activation of HIF1α or HIF2α, the 2 predominant HIF factors, may suppress GLS1 clustering. To test this notion, we investigated whether the suppression of GLS1 clustering by DMOG requires HIF1α or HIF2α. Knockdown or knockout (KO) of HIF1α (validated in Figure 6B and Supplemental Figure 6, E and F) did not block DMOG’s suppression of GLS1 clustering (Figure 6A and Supplemental Figure 6, B and D). In contrast, knockdown or KO of HIF2α (validated in Figure 6B and Supplemental Figure 6, E and F) abrogated the effect of DMOG (Figure 6A and Supplemental Figure 6, C and D). Similarly, pharmacological inhibition of HIF2α translation (validated in Supplemental Figure 6E) also prevented DMOG’s suppression of GLS1 clustering (Supplemental Figure 6, C and D). We conclude that HIF2α, but not HIF1α, suppresses GLS1 clustering. Interestingly, we noted that inhibitors of HIF2α transcriptional activation (HIF inhibitors VII and PT2385) did not reverse DMOG’s effect (Figure 6D), despite efficient inhibition of HIF2α transcriptional activity (Figure 6C), suggesting that the effects of HIF2α are not dependent on HIF2α transcriptional activity. Further supporting this conclusion, overexpression of a constitutively stabilized ΔTAD HIF2α mutant that lacks the C-terminal transcription activation domain (C-TAD, a region required for transcriptional activation of HIF target genes) rescued GLS1 clustering as efficiently as did transcriptionally active HIF2α (Supplemental Figure 6, G–I). Finally, we also observed that DMOG treatment suppressed glutamine-deprivation-induced cell death (Figure 6E), indicating a prosurvival role of HIF2α under these conditions. Together, these findings demonstrate that HIF2α, but not HIF1α, suppresses GLS1 clustering, and does so in a transcriptional activity–independent manner.

### GLS1 clustering in ccRCC is prevented by HIF2α.

**Figure 7:**
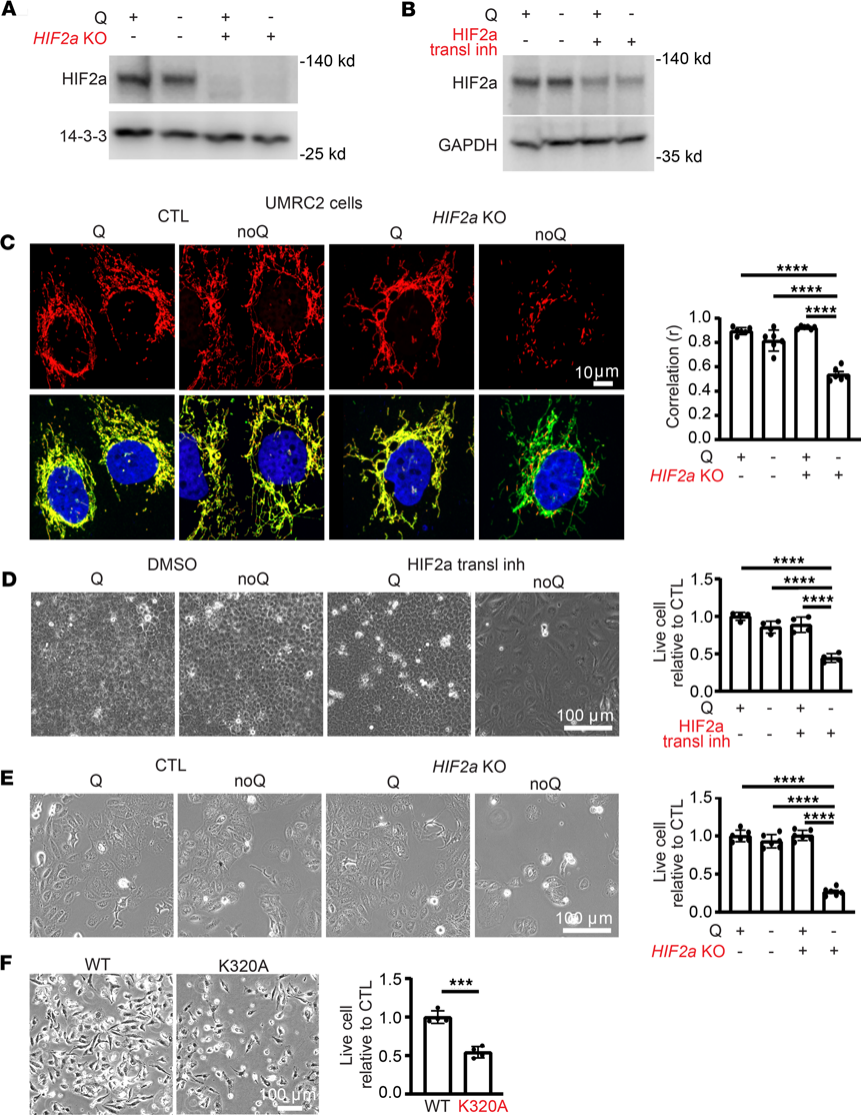
GLS1 clustering is prevented in UMRC2 cells in a HIF2α-dependent manner. (**A** and **B**) Western blotting analysis for the validation of *HIF2**α*** KO (**A**) and its translational inhibitor (**B**) in UMRC2 cells. (**C**) Resistance to noQ-induced GLS1 clustering in UMRC2 cells is reversed by *HIF2α* KO. (**D**) Resistance to noQ-induced cell death in UMRC2 cells is reversed by treatment with an inhibitor of HIF2α translation. (**E**) Resistance to noQ-induced cell death in UMRC2 cells is reversed by *HIF2α* KO. (**F**) Increased cell death in UMRC2 cells overexpressing the K320A mutant GLS1 compared with those overexpressing WT GLS. ***P < 0.001; ****P < 0.0001 by 1-way ANOVA followed by Bonferroni’s post hoc testing (**C**–**E**) or 2-tailed Student’s *t* test (**F**). Scale bars: 10 μm (**C**) and 100 μm (**D**–**F**).

Activation of HIF2α is observed in nearly all cases of ccRCC, usually as a result of *VHL* inactivation, and HIF2α is required for tumor development (35–37). In contrast, HIF1α, while also activated by loss of *VHL*, is dispensable for tumor development (36, 37). We thus hypothesized that GLS1 clustering would be suppressed in HIF2α-positive ccRCC cells, and furthermore that the inhibition of GLS1 clustering by HIF2α in these cells may contribute to cell viability and tumorigenesis, as suggested above (Figure 6E). Indeed, in contrast with all other cells we tested (Figures 1 and 2, and Supplemental Figure 3), both UMRC2 and 769-P cell lines, derived from human ccRCCs and which exhibit constitutive HIF2α activity (38) (Figure 7, A and B, and Supplemental Figure 7A), were resistant to GLS1 clustering under glutamine-deprived conditions (Figure 7C, Supplemental Figure 7C, and Supplemental Figure 8A). Moreover, this resistance was dependent on HIF2α as demonstrated by KO and siRNA knockdown (Figure 7, A and C, Supplemental Figure 7, A–C, and Supplemental Figure 8A). Coincident with the protection from GLS1 clustering, these cells were also protected from cell death induced by glutamine deprivation (Figure 7, D and E, Supplemental Figure 7D, and Supplemental Figure 8, B and C), akin to the protection seen with DMOG in other cells (Figure 6E), and suppressing HIF2α sensitized the cells to glutamine deprivation (Figure 7, D and E, Supplemental Figure 7D, and Supplemental Figure 8, B and C). Conversely, ectopic expression of the constitutively clustering GLS1 K320A mutant was sufficient to induce cell death in glutamine-deprived ccRCC cell lines (Figure 7F and Supplemental Figure 8D). These findings demonstrate that HIF2α-driven ccRCC cells actively suppress GLS1 clustering in a HIF2α-dependent fashion. Moreover, HIF2α-driven inhibition of GLS1 clustering is required to prevent cell death in low-glutamine environments, uncovering an important liability of these tumors.

To investigate HIF2α-dependent inhibition of GLS1 clustering in human RCC, we performed immunohistochemistry on primary human tissue samples, including normal kidney, ccRCC, and papillary RCC (pRCC) (Supplemental Figure 9A). We compared ccRCC and pRCC due to their distinct HIF biology; ccRCC is generally associated with *VHL* loss and constitutive HIF2α activation, while pRCCs generally do not have HIF2α activation, instead bearing mutations in several distinct pathways (39). Blinded immunohistochemical analyses revealed diffuse GLS1 staining seen in normal kidney samples and clear GLS1 clustering in pRCC samples (Supplemental Figure 9A), likely a response to low intratumor glutamine concentrations. In sharp contrast, almost no GLS1 clustering was seen in ccRCC samples (Supplemental Figure 9A), consistent with suppression of GLS1 clustering by activated HIF2α in the context of tumors lacking *VHL*. Together, these findings demonstrate that HIF2α-driven ccRCC cells actively inhibit GLS clustering, playing a key role in ccRCC biology, and uncover a potential liability in these lethal tumors.

### Promoting GLS1 clustering suppresses ccRCC tumor growth.

**Figure 8:**
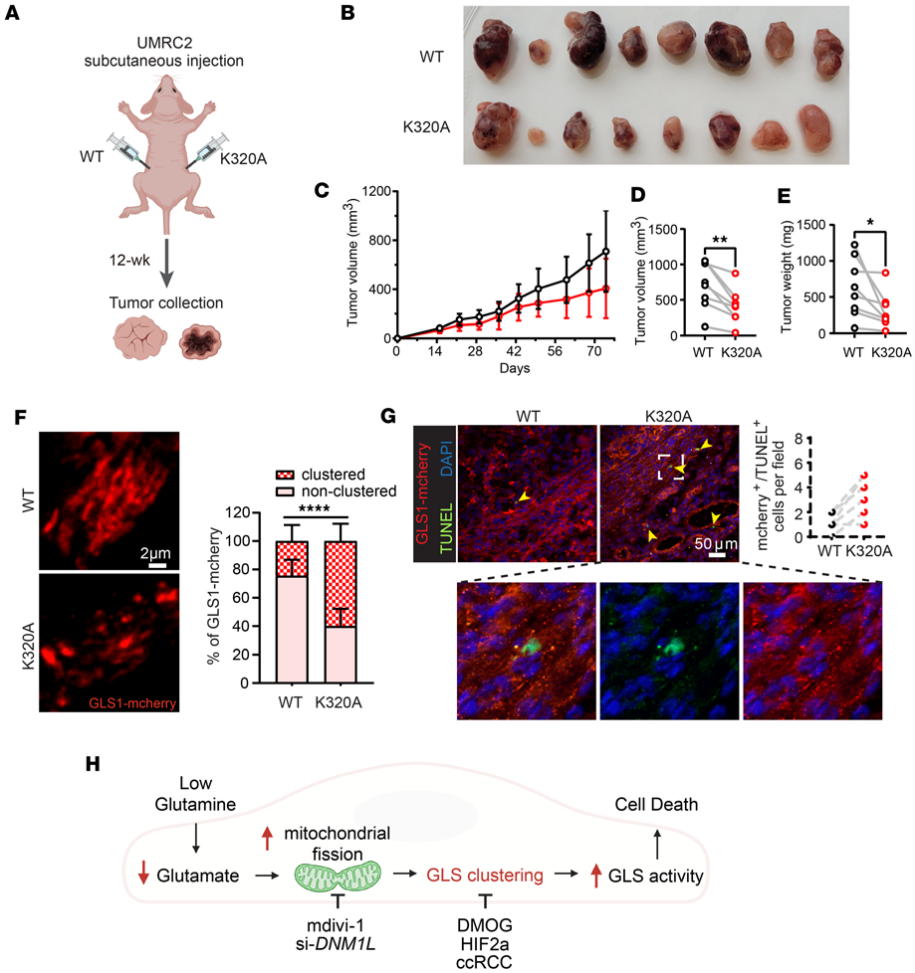
Promoting GLS1 clustering in UMRC2 suppresses tumorigenesis. (**A**) Schematic of the UMRC2 injection study. Each nude mouse received subcutaneous injections of 10 million UMRC2 cells overexpressing either WT GLS or K320A GLS1 into their left and right flanks, respectively. (**B**) Reduced tumor growth in K320A-overexpressing UMRC2. Photo of all dissected tumors: WT on the upper panel and K320A on the lower panel, *n* = 8 for each group. (**C**) Average tumor volume (mm^3^) in WT versus K320A GLS1–overexpressing UMRC2 during the 12-week monitoring period. Black circles: WT; red circles: K320A. (**D**) Pair-matched plot of tumor volume (mm^3^) in WT versus K320A GLS1–overexpressing UMRC2 in each mouse. (**E**) Plot of tumor weight (mg) in WT versus K320A GLS1–overexpressing UMRC2 in each mouse. (**F**) Confirmation of GLS1 clustering in K320A-overexpressing UMRC2 tumor. Left panel depicts the representative confocal images. Right panel shows the quantification of GLS1 clustering. *P < 0.05, **P < 0.01, ****P < 0.0001 by paired sample, 2-tailed Student’s *t* test. (**G**) Increased apoptosis in K320A-overexpressing UMRC2 tumor. Scale bars: 2 μm (**F**) and 50 μm (**G**). Inset magnification, ×6. (**H**) Schematic of the model.

To test this potential liability of GLS1 clustering in tumors in vivo, we used a subcutaneous xenograft model of ccRCC by injecting UMRC2 cells into the dorsal flanks of immunocompromised mice. Patient-derived xenografts from *VHL*-mutant ccRCC retain robust glutamine metabolism (both oxidative and reductive), supporting the relevance of the model (40). Animals received UMRC2 cells overexpressing either WT or K320A GLS1 on paired dorsal flanks, and tumor growth was monitored noninvasively for 10 weeks, followed by sacrifice and histological analysis (schematic in Figure 8A). Compared with WT, K320A GLS1–expressing tumors exhibited significantly reduced growth, with lower tumor volumes and weights (Figure 8, B–E). Immunohistochemistry confirmed robust GLS1 clustering in K320A GLS1–expressing tumors (Figure 8F), while TUNEL staining revealed increased apoptosis (Figure 8G), consistent with our in vitro findings (Figure 7F and Supplemental Figure 8D). The expression of WT and K320A GLS1 in UMRC2 cells was validated by Western blotting (Supplemental Figure 9B). [U-^13^C_5_]glutamine tracing demonstrated that enforced clustering significantly increased GLS activity, as shown by elevated production of labeled glutamate and αKG (Supplemental Figure 9, C–F). Consistent with these cell-based results, K320A tumors in vivo exhibited higher glutamate levels than WT tumors (Supplemental Figure 9, G and H), confirming that forced GLS1 clustering enhances enzymatic activity in the tumor context. To test the impact of GLS inhibition in vivo, we treated mice bearing WT and K320A-expressing UMRC2 tumors with CB-839 (telaglenastat), a clinically used GLS inhibitor. CB-839 effectively suppressed GLS1 enzymatic activity in both WT and K320A cells (Supplemental Figure 10A). In vivo, CB-839 treatment produced a modest trend toward delayed growth of WT tumors, consistent with the low baseline GLS1 activity in ccRCC, and had little additional effect on K320A tumors, which were already strongly growth suppressed (Supplemental Figure 10B). Of note, in this repeat xenograft study, we injected half the number of cells used in the study shown in Figure 8 (5 million vs. 10 million), rendering the difference between WT and K320A tumor growth even more striking (Supplemental Figure 10B), suggesting a possible threshold effect of tumor cell number on the manifestation of GLS1-clustering-dependent growth differences. Together, these findings demonstrate that forced GLS1 clustering enhances enzymatic activity, increases apoptosis, and suppresses ccRCC tumor growth. They further provide a mechanistic explanation for why GLS inhibitors show limited efficacy in ccRCC clinically; baseline GLS1 clustering and activity are already low due to HIF2α-imposed inhibition, leaving little room for pharmacologic inhibition to impact tumor growth.

## Discussion

In this study, we demonstrate that glutamine deprivation induces GLS1 clustering in various cell types, encompassing normal and cancer cells, and we elucidate the mechanisms by which GLS1 clustering occurs, as well as the enzymatic and functional consequences of GLS1 clustering. We propose a model (Figure 8H) whereby low extracellular glutamine reduces intracellular glutamate levels, leading to mitochondrial fission–mediated GLS1 clustering, which increases GLS1 enzymatic activity and promotes cell death. Moreover, we show that inhibition of this process actively occurs in ccRCC and is mediated by HIF2α, elucidating a new role for HIF2α, and uncovering a liability that could be leveraged to suppress tumor growth by promoting GLS1 clustering.

As noted above, ccRCC tumors are avid consumers of glutamine, but GLS inhibition, long entertained as a possible therapy for ccRCC, was not effective in a recent phase III PCDB-RCT (27). CB-839, the agent used in these trials, and its analog BPTES, inhibit GLS activity by allosteric prevention of the dimer-to-tetramer transition of GLS1 (41, 42) and of GLS1 supratetrameric formation (21, 22). Here we show that, in contrast, promoting GLS1 clustering, accompanied by markedly increased GLS1 enzymatic activity, also suppresses ccRCC tumor growth in a preclinical xenograft model. Recent work, performed in parallel to ours, has indicated similar effects of GLS1 clustering in suppressing glioma xenograft models (23). This raises the interesting possibility that, in certain contexts, reduced rather than increased GLS1 activity promotes ccRCC tumorigenesis. Tumors are notoriously heterogeneous, likely including areas of glutamine sufficiency but others of glutamine deprivation, where preventing GLS1 clustering may in fact be beneficial. Thus, our findings offer a possible explanation for why GLS inhibitors have not had therapeutic success so far.

HIF2α is likely the most important driver of ccRCC. As noted above, inhibition of HIF2α with belzufitan, a PT2385 analog, is now approved for treatment of ccRCC. However, clinical responses remain largely incomplete, and seen only in approximately 50% of patients (30), indicating the need for further understanding how HIF2α affects ccRCC biology. We now demonstrate here that HIF2α, but not HIF1α, suppresses GLS1 clustering and hyperactivation. This function for this oncogenic transcription factor likely critically contributes to tumor growth, because as we have shown, reversing this effect of HIF2α, i.e., constitutively forcing GLS1 clustering, reduces ccRCC tumor growth (Figure 7). Understanding mechanistically how HIF2α suppresses GLS1 clustering will be of great interest. Importantly, we found that PT2385 does not prevent HIF2α from suppressing GLS1 clustering. PT2385 blocks the formation of HIF2α-ARNT heterodimers, an obligatory event for HIF2α-mediated DNA binding and transcriptional activity. Thus, it appears that HIF2α blocks GLS1 clustering in a non-genomic fashion. The fact that PT2385 does not affect this function of HIF2α may in part explain the incomplete clinical efficacy of PT2385, and we predict, therefore, that there may exist an opportunity to synergize current HIF2α inhibition with additional stimulation of GLS1 clustering.

Why would too much GLS1 activity suppress tumor growth? GLS1 removes the γ-nitrogen amide group from glutamine, converting it to glutamate and releasing an ammonium ion. The γ-nitrogen amide group of glutamine is an indispensable donor of nitrogen for several essential cellular metabolic processes, including synthesis of asparagine, purine and pyrimidine nucleobases, and hexosamines. Glutamine is also critical for the synthesis of glutathione to defend against oxidative stress. Overactive GLS1 activity can thus be predicted, under certain circumstances, to deplete glutamine and to suppress these critical pathways. Indeed, the balance of glutamine utilization has been suggested to skew away from glutaminolysis and toward nucleotide synthesis during malignant progression (43). We have shown before that, to survive glutamine deprivation, endothelial cells activate macropinocytosis as an alternative source of asparagine and other NEAAs normally synthesized from glutamine (31). Additionally, Jiang et al. demonstrated in human embryonic brain cells that asparagine supplementation rescues cell death induced by low glutamine (23). Thus, under some circumstances, GLS1 aggregation in response to glutamine deficiency may be a maladaptive response that skews glutamine use toward glutaminolysis, perhaps to optimize anaplerosis, but at the expense of use of its γ-nitrogen amide for other critical cell functions. It is also possible that the redistribution of GLS1 to specific submitochondrial compartments causes metabolic channeling to specific pathways, in a way that is deleterious to cellular viability. Finally, clustered GLS1 may also have proapoptotic activity that is independent of its enzymatic activity. Suggestive of this possibility, Jiang et al. showed that αKG blocks clustering and rescues cell death (despite not providing γ-nitrogen amide groups) in human embryonic brain cells, but αKG does not do so in cells expressing the constitutively clustered K320A mutant (23).

How is GLS1 clustering regulated? Ferreira et al. (22) and, more recently, Adamoski et al. (21) using cryogenic electron microscopy (EM), demonstrated that inorganic phosphate (Pi) allosterically activates GLS1 and promotes enzyme filamentation. Adamoski et al. (21) further used EM to show that intramitochondrial GLS1 clustering seen in intact cells corresponded to GLS1 filamentation, as seen in vitro. To what extent Pi regulates this process under physiological conditions, however, is not clear. Glutamine deprivation does not appear to alter intracellular Pi levels (23). Instead, we found, as did Jiang et al. (23), that GLS1 polymerization is regulated by intracellular glutamate levels, within physiological ranges. Interestingly, Hans Krebs first noted in 1935 that glutaminolysis in kidney extracts (i.e., GLS1), but not liver extracts (i.e., GLS2), was exquisitely sensitive to inhibition by glutamate, and he hypothesized competitive inhibition between glutamine and glutamate (44). Subsequent biochemical work confirmed this hypothesis, showing competition that favored glutamate binding at a [Pi] of approximately 10 mM (a typical intramitochondrial concentration), while favoring glutamine binding at high [Pi] (45). Precisely how glutamate regulation occurs will require structural studies akin to those carried out by Adamoski et al. (21).

With respect to regulation of GLS1 clustering, we also show that glutamine deprivation triggers a transient cycle of mitochondrial fission/fusion that is required to enable clustering of GLS1 into distinct intramitochondrial puncta. The dynamics of mitochondrial fusion and fission are pivotal for maintaining mitochondrial quality control. Typically, mitochondrial fragmentation occurs under conditions of nutrient excess, whereas nutrient starvation induces mitochondrial elongation (46). Consistent with this paradigm, studies have shown that glutamine deprivation leads to mitochondrial elongation within 4–6 hours (32, 33). In contrast with these observations, our study reveals a distinctive pattern; immediately after glutamine starvation, within the initial 1–2 hours, mitochondria undergo excessive fission, followed by resumption of mitochondrial networks by fusion. Prior studies may have missed this initial time window. Strikingly, we found that this rapid fission/fusion process is essential for GLS1 clustering, while other mitochondrial proteins remain unaffected after glutamine deprivation. This specificity may be explained by the intrinsic ability of GLS1 to polymerize, making it uniquely responsive to mitochondrial dynamics. The fission/fusion cycle could provide the structural remodeling needed for self-assembling GLS1 oligomers to reach a critical size, thereby enabling clustering. Alternatively, mitochondrial remodeling may alter the local metabolic environment, e.g., by altering intramitochondrial glutamate concentrations, in ways that promote GLS1 oligomerization. Further studies will be needed to dissect how mitochondrial dynamics confer this selective effect of GLS1.

In summary, we show here that glutamine deprivation induces GLS1 clustering in various cell types, and we reveal several mechanistic aspects of this process. Importantly, we show that HIF2α blocks GLS1 clustering in ccRCC, thereby promoting tumor growth. The work elucidates multiple aspects of glutamine handling, including what we believe is a novel connection between glutamine handling and HIF2α, the predominant driver of ccRCC, thus uncovering a potential therapeutic avenue to synergize with HIF2α inhibition in the treatment of ccRCC.

## Methods

### Sex as a biological variable.

Our study exclusively examined female mice. It is unknown whether the findings are relevant for male mice.

### Cell culture.

293T, HeLa, HCT116, HepG2, C2C12, UMRC2, and 769-P cells were purchased from ATCC and cultured in DMEM (Gibco, 11995056) supplemented with 10% FBS. HUVECs were purchased from Lonza and cultured in EBM2 containing EGM supplements (Lonza, CC-3162) with 10% FBS. Glutamine deprivation studies were done using DMEM that contains no glutamine (Gibco, 31053028) supplemented with 10% dialyzed FBS (HyClone).

For siRNA and DNA transfection, cells were kept in serum-free Opti-MEM media (Gibco, 31985062) for 6 hours of transfection duration, after which they were refreshed with their complete media. All siRNAs were used at 10 nM concentration and were obtained from Sigma-Aldrich: human si-GLUL (SASI_Hs02_00307974), human si-SLC7A11 (SASI_Hs02_00345461), human si-DNM1L (SASI_Hs02_00340086), human si-HIF1α (SASI_Hs02_00332063), and human si-HIF2α (SASI_Hs01_00019152). For the HIF2α overexpression experiment, HUVECs were transfected with either a constitutively stabilized HIF2α triple mutant (HIF2α-TM; Addgene, 44027) (47), which carries proline-to-alanine (P405A), proline-to-valine (P530V), and asparagine-to-alanine (N851A) substitutions that stabilize the protein and maintain its transcriptional activity, or with an HIF2α-ΔC-TAD (47) construct lacking the C-terminal transactivation domain (amino acids 821–874), which abolishes HIF2α’s transcriptional activation function. Genetic KOs of *HIF1α* and *HIF2α* in HUVECs and UMRC2 cells were made using CRISPR/Cas9 technology. Specifically, gRNA sequences targeting *HIF1α* (seq1: TGGCTCATATCCCATCAATT; seq2: ACAGTAACCAACCTCAGTGT; seq3: TGAACATAAAGTCTGCAACA; seq4: GATAATGTGAACAAATACAT) and *HIF2α* (seq1: ACCGGATGCTCGCAAAGCAT; seq2: TGTTCTCGGAGTCTAGCGCA; seq3: TAGCCACACAGACTATTGTG; seq4: CAAGTTCATGGGACTTACAC) were cloned into the lentiCRISPRv2 plasmid. Lenti-X 293T cells (Takara, 632180) were transfected with plasmids pMD2.G (Addgene, 12259), psPAX2 (Addgene, 12260), and cloned lentiCRIPSRv2 for virus production. KO cells were obtained after viral infection and puromycin selection. Confirmation of siRNA/DNA-mediated genetic manipulation/KO was determined using multiple different methods, including qPCR, Western blotting, or ICC.

Cell death was assessed using a commercial kit (Thermo Fisher Scientific, V13241) according to the manufacturer’s instructions. The following drugs and chemicals were used at the concentrations indicated here or in the figures: dimethyl-αKG (Sigma-Aldrich, 349631), NH_4_Cl (Sigma-Aldrich, A9434), L-glutamate (Sigma-Aldrich, G8415), MSG (Sigma-Aldrich, G5889), cycloheximide (2 μg/mL; Sigma-Aldrich, 239765), EGCG (Sigma-Aldrich, E4143), AOA (Sigma-Aldrich, C13408), SASP (Sigma-Aldrich, S0883), mdivi-1 (20 μM; Sigma-Aldrich, M0199), DMOG (Sigma-Aldrich, D3695), HIF2α translation inhibitor (Sigma-Aldrich, 400087), HIF inhibitor VII (Sigma-Aldrich, 5043790001), CB-839 (Selleck, S7655), and PT2385 (MedChemExpress, HY-12867).

For the isotope tracer assay using [U-^13^C_5_]glutamine, UMRC2 cells were grown in 6-well plates and incubated with growth medium (DMEM without glutamine; Gibco, 31053028) containing [U-^13^C_5_]glutamine (Cambridge Isotope Laboratories, CLM-1822-H-0.1; 2 mM), 10% dialyzed FBS (Cytiva, SH30079.02), and 1% penicillin/streptomycin. After incubation, culture media were harvested, and cells were washed with 37°C saline and lysed immediately for LC-MS.

### ICC.

Cells were plated onto glass coverslips and subjected to siRNA transfection, drug treatment, and/or glutamine starvation as indicated in the figures. Cells were then fixed with 3.7% paraformaldehyde, washed, and permeabilized with 0.3% Triton X-100. After blocking with 2% BSA, samples were incubated with primary antibodies overnight. Primary antibodies used for the ICC were GLS1 (Abcam, ab156876), COXIV (Cell Signaling Technology [CST], 11967 and 4850), GLUD (Novus, NBP1-68846), PDH (Abcam, ab110333), HADHA (Abcam, ab203114), COX4-I1 (R&D Systems, AF5814), Lamp1 (DSHB, H4A3-c), calnexin (Thermo Fisher Scientific, MA3-027), golgin (Thermo Fisher Scientific, 14-9767-82), Tom20 (Santa Cruz Biotechnology, sc-11415), and CS (Sigma-Aldrich, SAB2702186). After washing with PBS, the samples were incubated with secondary antibodies for 2 hours at room temperature. All secondary antibodies used are conjugated with Alexa Fluor (Invitrogen) dyes — 488, 555, or 647. Finally, the samples were washed and mounted onto glass slides using ProLong Diamond Antifade Mountant (Invitrogen) for imaging under a wide-field or confocal microscope (Leica), as described in the figure legends.

### Correlation coefficient (r) measurement.

Pearson’s correlation coefficient was measured using CellProfiler version 4.2.5 (https://cellprofiler.org/previous-releases) to assess the degree of overlap between GLS1 and mitochondrial protein staining. The obtained values for representative images are indicated in yellow in each figure.

### Mitochondrial fractionation and Western blotting.

Mitochondrial subfractionation was performed by using the Mitochondria Isolation Kit (Thermo Fisher Scientific, 89874) following the manufacturer’s instructions. The mitochondrial fraction along with total cell lysate and cytosolic fractions were lysed in RIPA buffer that contained cOmplete Mini Protease Inhibitor Cocktail (Roche) and phosphatase inhibitor (PhosSTOP, Roche). Protein concentration was measured by BCA protein assay kit (Thermo Fisher Scientific) and the samples were then boiled in Laemmli buffer and loaded into 4%–20% gradient gels (Bio-Rad), transferred to PVDF membranes (Millipore), and analyzed by immunoblotting. The following primary antibodies were used: GLS1 (Abcam, ab156876), CS (Sigma-Aldrich, SAB2702186), TIMM23 (Abcam, ab116329), DRP1 (Proteintech, 12957-1-AP), HIF1α (Novus Bio, NB100-296), HIF2α (CST, 7096s), MYC-tag (CST, 2278s), mCherry (CST, 43590), 14-3-3 (CST, 8312), and GAPDH (CST, 5174). Secondary antibodies that are conjugated with HRP were purchased from CST. Signal was detected using the ECL system (ImageQuant LAS 4000, Amersham Biosciences, GE Healthcare) according to the manufacturer’s instructions.

### qPCR.

mRNA isolation and cDNA synthesis were done by using the TurboCapture mRNA Kit (QIAGEN) according to the manufacturer’s instructions. qPCR was performed on the CFX384 Bio-Rad Real-Time PCR Detection System using SYBR Green. Sequences of the primers used in this study were as follows: *HIF1a* (forward, 5′-TATGAGCCAGAAGAACTTTTAGGC-3′, reverse, 5′-CACCTCTTTTGGCAAGCATCCTG-3′), *HIF2a* (forward, 5′-CTGTGTCTGAGAAGAGTAACTTCC-3′, reverse, 5′-TTGCCATAGGCTGAGGACTCCT-3′), *LDHA* (forward, 5′-TTGACCTACGTGGCTTGGAAG-3′, reverse, 5′-GGTAACGGAATCGGGCTGAAT-3′), *PGK1* (forward, 5′-GACCTAATGTCCAAAGCTGAGAA-3′, reverse, 5′-CAGCAGGTATGCCAGAAGCC-3′), *HK2* (forward, 5′-GGGACAATGGATGCCTAGATG-3′, reverse, 5′-GTTACGGACAATCTCACCCAG-3′), *ENO1* (forward, 5′-TGGTGTCTATCGAAGATCCCTT-3′, reverse, 5′-CCTTGGCGATCCTCTTTGG-3′), *ANGPT2* (forward, 5′-ATTCAGCGACGTGAGGATGGCA-3′, reverse, 5′-GCACATAGCGTTGCTGATTAGTC-3′), *PDK1* (forward, 5′-CTATGAAAATGCTAGGCGTCTGT-3′, reverse, 5′-TGGGATGGTACATAAACCACTTG-3′), *VEGFA* (forward, 5′-TTGCCTTGCTGCTCTACCTCCA-3′, reverse, 5′-GATGGCAGTAGCTGCGCTGATA-3′), *DLL4* (forward, 5′-TGGGTCAGAACTGGTTATTGGA-3′, reverse, 5′-GTCATTGCGCTTCTTGCACAG-3′), and *VEGFR2* (forward, 5′-GGAACCTCACTATCCGCAGAGT-3′, reverse, 5′-CCAAGTTCGTCTTTTCCTGGGC-3′).

### Determination of media glutamine concentration.

Metabolites were extracted from 100 μL of culture medium with 1 μL of 4N hydrochloric acid, immediately followed by addition of 400 μL of dry-ice-cold analytical-grade methanol. Each sample was spiked with 18.75 nmol of L-norvaline as an internal standard, provided in the methanol. Samples were incubated on dry ice for 15 minutes and then centrifuged at 21,000*g* for 15 minutes at 4°C. Supernatants containing soluble metabolites were transferred to new tubes and dried under vacuum in a Speedvac microcentrifuge concentrator. In a fume hood, dried samples were resuspended in 100 μL of room-temperature 1:1 (vol/vol) analytical-grade acetonitrile and *N*-methyl-*N*-(*tert*-butyldimetylsilyl)trifluoroacetamide (MtBSTFA; Regis Technologies, 1-270242-200), and were heated on a 70°C heating block for 90 minutes. Then samples were cooled to room temperature (~5 minutes), centrifuged at 13,000*g* for 5 minutes, and the supernatant was transferred to GC-MS vials with polypropylene inserts for small volume samples. One microliter of the sample was injected via automatic liquid sampler (Agilent 7693A) into an Agilent 7890B gas chromatograph (GC) coupled with an Agilent 5977B mass selective detector (MSD) (Agilent Technologies). The GC was operated in splitless injection mode with helium as the carrier gas at a flow rate of 1.2 mL/min. The GC column was a 30 m × 250 μm × 0.25 μm HP-5ms Ultra Inert column (Agilent, 19091S-433UI). The inlet temperature was 250°C, and after 3 minutes at 100°C, the oven temperature program was increased as follows: 4°C/min to 230°C then 20°C/min to 300°C and hold 5 minutes. The transfer line temperature was 250°C, and the MSD source and quadrupole temperatures were 230°C and 150°C, respectively. After a 6-minute solvent delay, the MSD was operated in electron ionization mode and scan mode with a mass range of 50–550 AMU at 2.9 scans/s. Agilent MassHunter Qualitative Analysis software (B.07.00) was used for visualization of chromatograms. A standard curve of glutamine in culture medium was used to determine glutamine concentrations.

### GLS1 activity assay.

For the experiment in Figure 5, HUVECs were preconditioned in 2 mM Q–containing versus noQ media for 24 hours. Subsequently, 2 mM uniformly labeled [U-^13^C_5_]glutamine (Cambridge Isotope Laboratories) was introduced for the indicated time, and ^13^C-glutamate was measured as a readout of GLS1 activity. Briefly, intracellular metabolites were extracted by aspirating the medium and quickly adding 80% methanol prechilled at –80°C. Following a 20-minute incubation on dry ice, the resultant mixture was scraped, collected into a tube, and centrifuged at 10,000*g* for 5 minutes. The supernatants were dried under nitrogen gas and analyzed using reversed-phase ion-pairing chromatography coupled with negative mode ESI high-resolution mass spectrometry on a stand-alone orbitrap. EI-MAVEN was used for peak picking (https://github.com/ElucidataInc/ElMaven/releases; commit ID 263cfb7), and Accucore (https://github.com/XiaoyangSu/AccuCor; commit ID 1855199) was used for natural isotope correction.

### LC-MS.

Culture medium samples were centrifuged at 2000*g* for 5 minutes at 4°C to remove cell debris. Ten microliters of medium was extracted using 100 μL prechilled (–20°C) lysis buffer (40% methanol, 40% acetonitrile, 20% water). For analysis of cellular metabolites, cells were cultured in a 6-well plate and lysed using 200 μL prechilled lysis buffer. For LC-MS of tumors, frozen samples stored at –80°C were ground at liquid nitrogen temperature with a Cryomill (Retsch). Tissue powder was then weighed (~20 mg) and extracted with prechilled lysis buffer (~40× tissue weight, a concentration of 25 mg/mL). All LC-MS samples were then centrifuged twice at 16,000*g* for 10 minutes at 4°C. The final supernatant was transferred to LC-MS tubes for analysis. Targeted measurements of glutamine, glutamate, and αKG were achieved on a quadrupole orbitrap mass spectrometer (Thermo Fisher Scientific, Q Exactive) coupled to hydrophobic interaction chromatography (HILIC) via electrospray ionization. LC separation was performed on an XBridge BEH Amide Column (2.1 × 150 mm, 2.5 μM particle size, and 130 Å pore size; Waters Corporation) using a gradient of solvent A (water/acetonitrile [95:5] with 20 mM ammonium acetate and 20 mM ammonium hydroxide, pH 9.45) and solvent B (acetonitrile). The following gradient was used: 0 minutes, 90% B; 2 minutes, 90% B; 3 minutes, 80% B; 5 minutes, 80% B; 6 minutes, 75% B; 7 minutes, 75% B; 8 minutes, 70% B; 9 minutes, 70% B; 10 minutes, 50% B; 11 minutes, 50% B; 12 minutes, 40% B; 14 minutes, 40% B; 15 minutes, 90% B; 20 minutes, 90% B. The injection volume was 5 μL and the autosampler temperature was set at 4°C. The total running time was 20 minutes at a flow rate of 150 μL/min. The data were generated using negative ion mode with a scan range of 65–835 *m*/*z* and resolution of 140,000, and normalized by cell number and total ion counts.

### Mitochondrial fragmentation count as a mitochondrial fission index.

Mitochondrial fragmentation count was calculated by counting non-contiguous mitochondrial particles and dividing by the number of pixels that comprise the mitochondrial network (48).

### WT versus K320A GLS1 overexpression.

WT or K320A GLS1 was overexpressed in HeLa, UMRC2, and 769-P cells using a retroviral infection system. GLS1-myc-mCherry sequences were cloned into the retroviral pLHCX plasmid (Addgene, 44239) and transfected into HEK293T cells for virus generation. Virus-containing media were filtered and used for transduction of HeLa, UMRC2, and 769-P with WT or K320A GLS1.

### Human kidney sample immunohistochemistry and quantification.

Kidney cancer tissue microarray slides (T071b, KD809, and KD2085) were purchased from TissueArray. The slides were deparaffinized in xylene, rehydrated through a graded ethanol series, and quenched in 0.3% hydrogen peroxide/methanol for 15 minutes. For antigen retrieval, slides were boiled for 20 minutes in 10 mM sodium citrate (pH 6.0). Sections were blocked with 5% goat serum/1% BSA/0.5% Tween 20 for 1 hour and then incubated with primary antibody against GLS (1:200; Abcam, ab156876) diluted in blocking buffer overnight at 4°C. Following primary antibody incubation, slides were treated with biotinylated secondary antibodies for 1 hour, followed by ABC solution (Vector Laboratories) for 30 minutes. Staining was developed with 3,3′-diaminobenzidine (Vector Laboratories). Slides were counterstained with hematoxylin, dehydrated, and mounted with Permount (Thermo Fisher Scientific). Stained slides were scanned using a Leica Aperio Slide Scanner at the Penn Molecular Pathology & Imaging Core. Punctate GLS expression was confirmed by visual inspection at ×400 magnification. Slides showing abundant GLS puncta were considered as clustered, while those with few puncta or low GLS expression were scored as non-clustered. Images were analyzed in a binary manner, scored as either clustered or non-clustered, and were quantified by the same scientist in a consistent manner.

### Tumorigenesis assay.

Female NIH-III nude mice (Charles River Laboratories, strain code: 201) that were 6–8 weeks old were subcutaneously injected in each flank with 10 million UMRC2 cells overexpressing either WT GLS1 or K320A GLS. Each mouse received both a WT and K320A GLS1 tumor on separate flanks, ensuring each mouse served as its own control for a pair matched comparison. Cells were resuspended in ice-cold PBS and combined 1:1 with Matrigel (BD Biosciences, 356234) for a final volume of 200 μL per injection. Tumor volumes were recorded at the indicated time points using caliper measurements, calculated by the formula *V* = (π/6) × *L* × *W*^2^, where *L* was the longer measurement and *W* was the shorter measurement. Tumors were harvested at the 12-week time point for weight and immunohistochemical analyses. For the experiments involving GLS inhibition, female nude mice (Charles River Laboratories, strain code: 201) aged 9–11 weeks were used and injected subcutaneously with 5 million UMRC2 cells in the flank. Tumor growth was monitored weekly to assess the best timing for the treatment with CB-839/vehicle. Mice were regrouped based on tumor volumes and body weights using RandoMice tool (49) before treatment. CB-839 (Selleck, S7655; 200 mg/kg) was prepared in the vehicle (20% [2-hydroxypropyl]-β-cyclodextrin, 10 mM citrate; pH 2.0) and administrated twice a day by oral gavage for 2 weeks. For tissue immunohistochemistry, following embedding in OCT (Sakura), tumor section and staining were performed as previously reported (50). In brief, tumors were sectioned at 5 μm. Slides were treated with 0.2% Triton X-100 in PBS and incubated in blocking solution (1% BSA, 5% goat serum in PBS) for 1 hour at room temperature. Primary antibodies anti-mCherry (Abcam, ab205402; 1:200) and anti-COX4 (CST, 11967S; 1:100) were used for overnight incubation at 4°C, followed with staining with secondary antibodies (Invitrogen, Alexa Fluor 555 conjugated and Alexa Fluor 488 conjugated, 1:400) or TUNEL enzymatic mixture (Roche, 11684795910) according to the manufacturers’ instructions. Slides were mounted using ProLong Gold antifade reagent with DAPI (Invitrogen, P36935) and examined by Zeiss LSM 710 confocal microscope. TUNEL/mCherry double-positive cells were quantified manually in each image for apoptosis analysis.

### Statistics.

Statistical comparisons among study groups were performed using either a 2-tailed Student’s *t* test or 1-way ANOVA followed by Bonferroni’s post hoc testing. A *P* value of less than 0.05 was considered statistically significant. All data are presented as mean ± SD. Results from cell culture experiments are representative of a minimum of 3 independent experiments.

### Study approval.

All mouse experiments were performed according to procedures approved by the University of Pennsylvania Institute for Animal Care and Use Committees (Philadelphia, Pennsylvania).

### Data availability.

All numerical data appearing in this article are included in the Supporting Data Values file.

## Author contributions

Boa Kim and WZ led the studies and were directly involved in most experiments. Boa Kim and ZA oversaw the studies. WZ, SMD, NJC, YJ, Boyoung Kim, SB, CB, MCN, CJ, and Boa Kim conducted experiments and acquired data. WZ, MCS, ZA, and Boa Kim interpreted data. WZ, ZA, and Boa Kim wrote the manuscript. All authors discussed the results and commented on the manuscript.

## Funding support

This work is the result of NIH funding, in whole or in part, and is subject to the NIH Public Access Policy. Through acceptance of this federal funding, the NIH has been given a right to make the work publicly available in PubMed Central.

- National Cancer Institute grants F30CA271654 (to NJC) and F31CA261041 (to MCN).
- National Research Foundation of Korea Basic Science Research Program grant RS-2024-00412498, funded by the Korean Ministry of Education (to Boyoung Kim).
- National Heart, Lung, and Blood Institute grants HL167014 (to ZA) and R56HL162660 (to Boa Kim).
- Department of Defense grant KC220099 (to ZA).
- The Ludwig Cancer Research center (to ZA).
- American Heart Association grant 24CDA1264317 (to Boa Kim).
- National Institute on Alcohol Abuse and Alcoholism grant AA029124 (to CJ).
- National Cancer Institute Cancer Center Core Support Grant P30 CA016086 (to the University of North Carolina Lineberger Comprehensive Cancer Center), as partial support for the Microscopy Services Laboratory, Department of Pathology and Laboratory Medicine.

## Supplementary Material

Supplemental data

Unedited blot and gel images

Supporting data values

## Version 1 10/30/2025

In-Press Preview

## Version 2 12/08/2025

Electronic publication
