# Supplementary material for: HIF2α inhibits glutaminase clustering in mitochondria to sustain growth of clear cell renal cell carcinoma
Source: JCI Insight. 2025 Oct 30;10(23):e182711. doi: 10.1172/jci.insight.182711 (PMC12890500; doi:10.1172/jci.insight.182711)
Supplement: Supplemental data [file jciinsight-10-182711-s126.pdf]

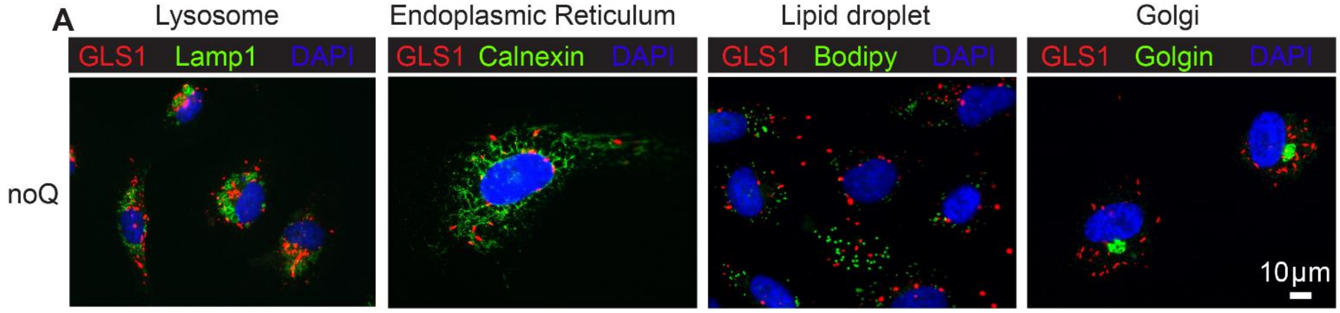

SFigure1. Clustered GLS1 does not co-localize with non-mitochondrial organelles.

: Co-staining of GLS1 with markers of various intracellular organelles in HUVECs upon 24-hour culture in noQ media: lysosome (Lamp1), endoplasmic reticulum (Calnexin), lipid droplet (BODIPY), or golgi (golgin).

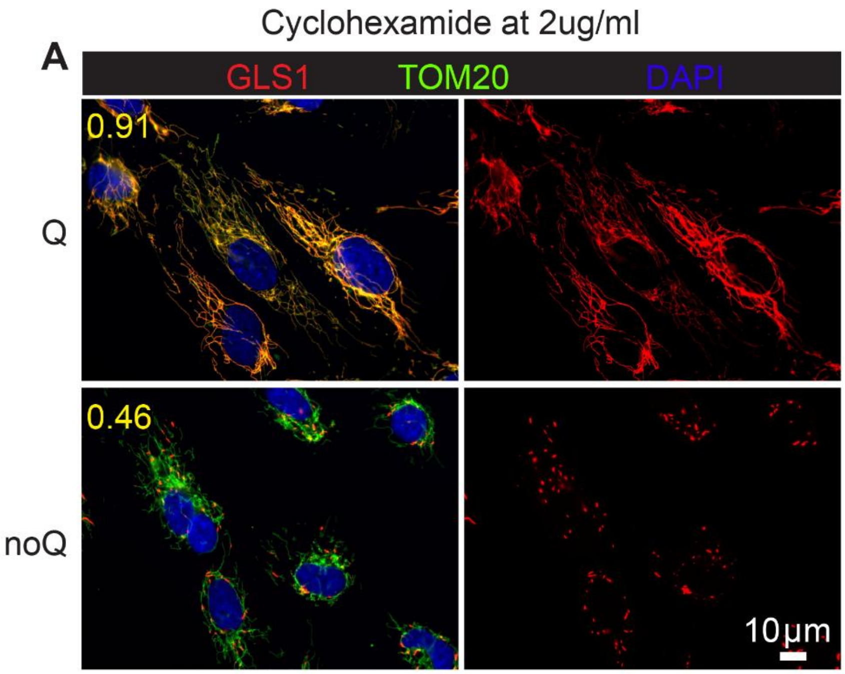

SFigure2. Existing, but not the newly synthesized, GLS1 cluster upon glutamine deprivation.
: ICC of GLS1 (red) and TOM20 (green) in HUVECs after a 24-hour culture in Q vs. noQ media in the presence of cycloheximide (2µg/ml).

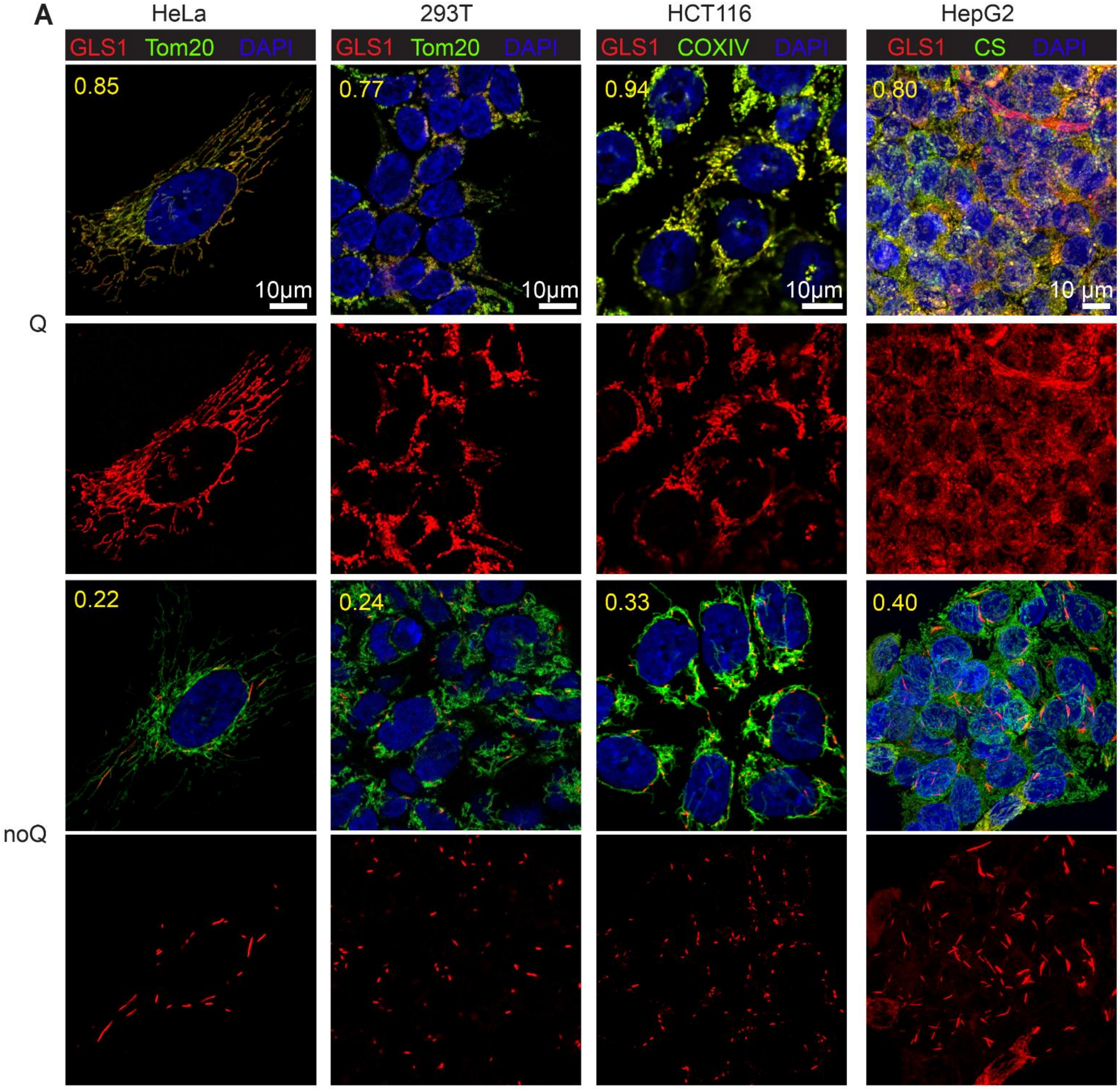

SFigure3. Glutamine deprivation-induced GLS1 clustering occurs ubiquitously in various cell types.
: ICC of GLS1 (red) and mitochondrial marker proteins (Tom20, COXIV, or CS in green) in various cell types: HeLa, 293T, HCT116 and HepG2.

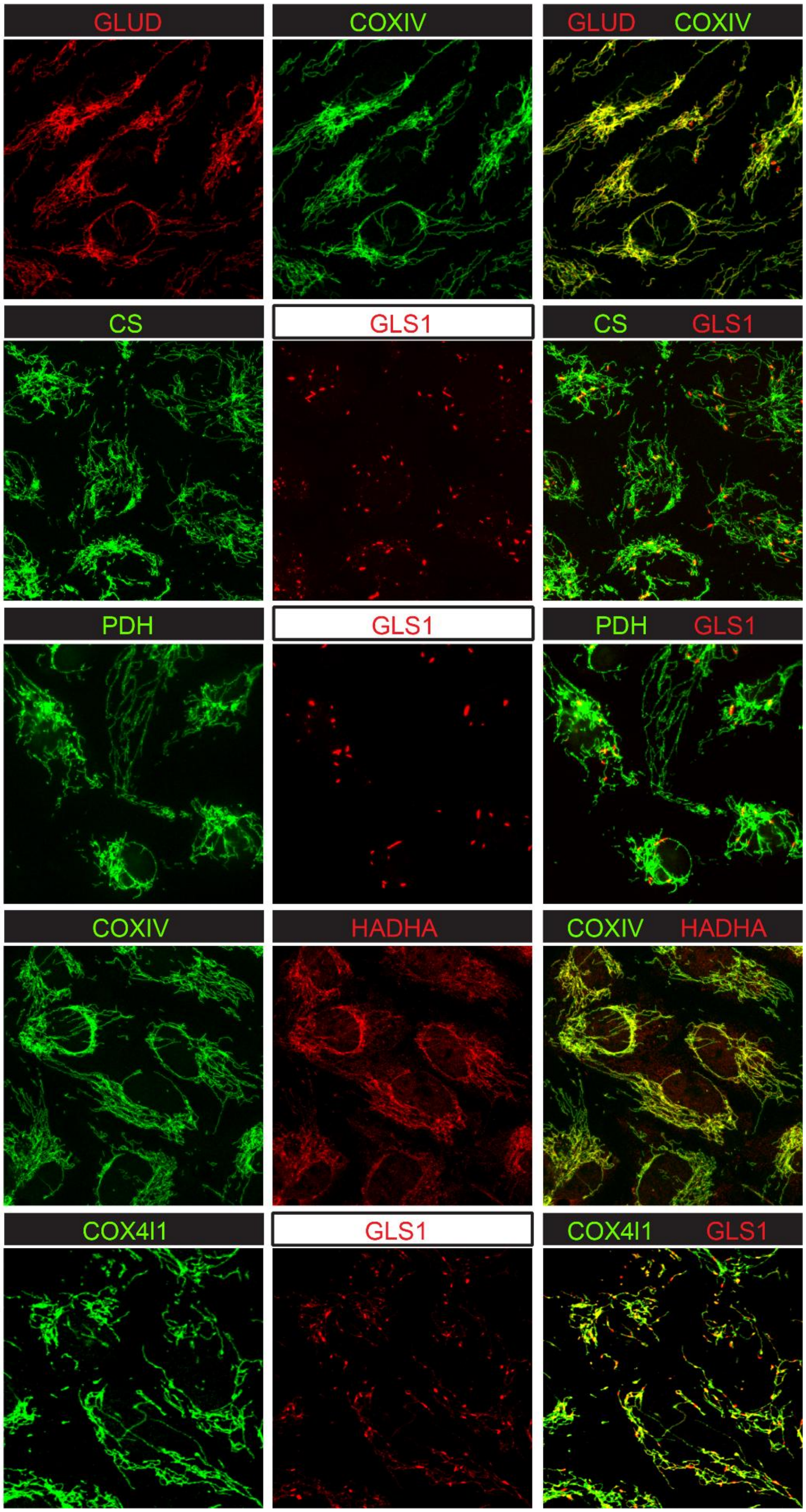

SFigure4. Clustering upon glutamine deprivation occurs uniquely to GLS1.

: ICC of various mitochondrial proteins in HUVECs after 24-hour culture in noQ media: GLUD, COXIV, CS, PDH, HADHA, and COX4l1.

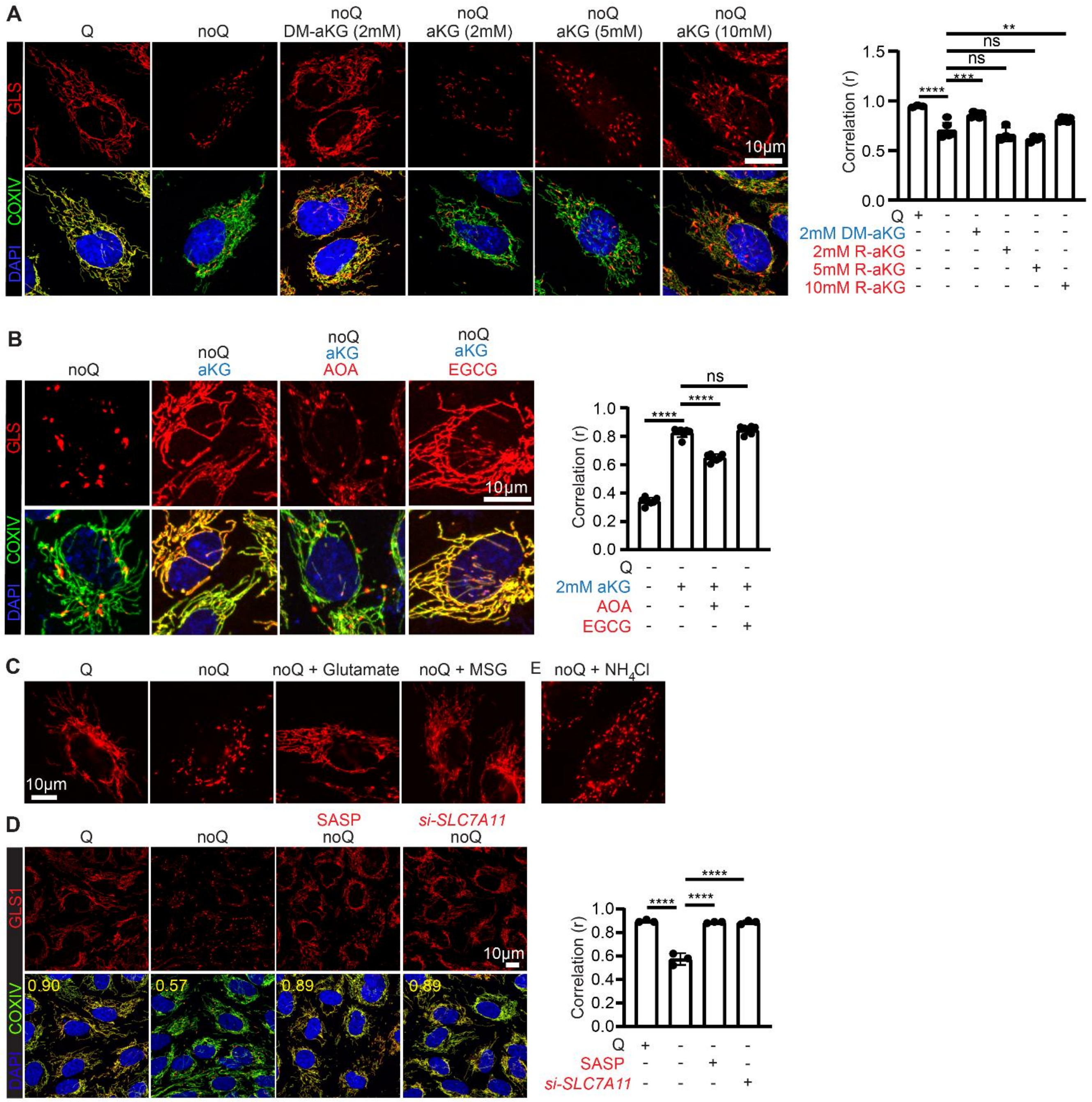

SFigure5. Restoration of glutamate level restores GLS1 clustering.

A. ICC demonstrating the rescued GLS1 clustering by DM-aKG and aKG in HUVECs. ** $p < 0.01$, *** $p < 0.001$, **** $p < 0.0001$ and ns (not significant, $p \geq 0.05$) by 1-way ANOVA. **B**. ICC showing the reversal of the aKG-induced GLS1 redistribution by AOA or EGCG. **** $p < 0.0001$ and ns by 1-way ANOVA. **C**. Rescued GLS1 clustering by the supplementation of glutamate or monosodium glutamate (MSG) in noQ for 6 hours. **D**. Rescue of GLS1 clustering by chemical inhibition (by SASP treatment at 300μM) or siRNA knockdown of SLC7A11. **** $p < 0.0001$ by 1-way ANOVA. **E**. No effect of supplementation of ammonia by NH_4Cl on GLS clustering.

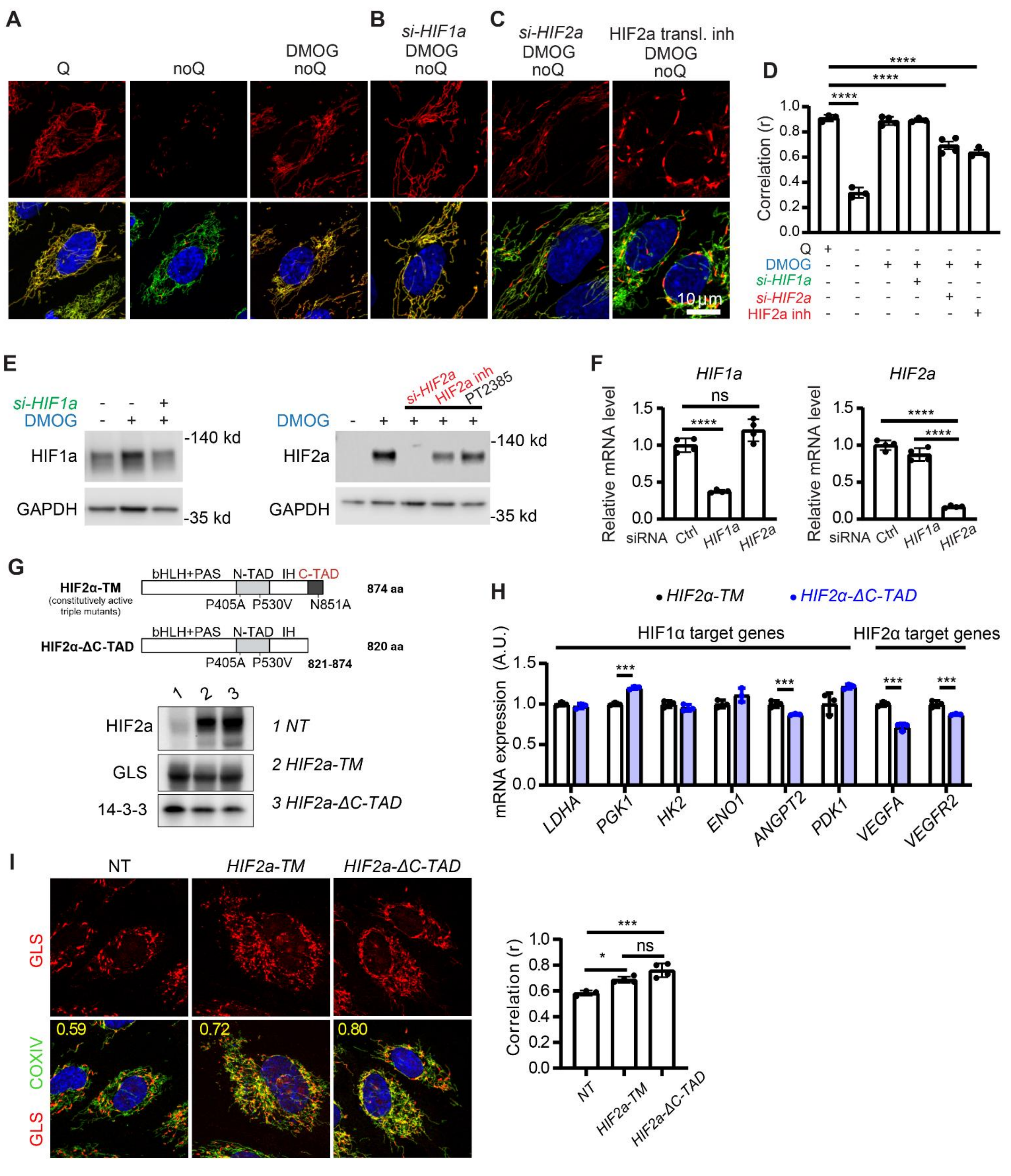

SFigure6. HIF2α protein but not its transcriptional activity inhibits GLS1 clustering.

A. Rescue of GLS1 clustering by DMOG treatment in HUVECs. **B**. No reversal of the DMOG-induced GLS1 redistribution by knockdown of HIF1α. **C**. Reversal of the DMOG-induced GLS1 redistribution by knockdown of HIF2α or inhibition of HIF2α translation. **D**. Correlation coefficient (r) for the conditions in **A, B** and **C**. **** $p < 0.0001$ by 1-way ANOVA. **E**. Western blotting analysis showing the validation of siRNA and chemical compounds on the expression of HIF1α (left panel) or HIF2α (right panel) in HUVECs. **F**. qPCR analysis showing the validation

of si_HIF1α and si_HIF2α in HUVECs. **** $p < 0.0001$ and ns by 1-way ANOVA. **G**. Schematic of HIF2α constructs (top: constitutively active HIF2α triple mutant; bottom: ΔC-TAD mutant lacking the C-terminal transactivation domain) and validation of their overexpression in HUVECs by western blotting. The ΔC-TAD mutant is ~5 kDa smaller than full-length HIF2α, consistent with the expected size difference (874 aa vs. 820 aa). NT, not transfected. **H**. qPCR analysis showing the suppression of HIF2α-ΔC-TAD on the mRNA expression of HIF2α target genes. *** $p < 0.001$ by 1-way ANOVA. **I**. ICC showing the inhibition of GLS1 clustering by the overexpression of HIF2α and HIF2α-ΔC-TAD. * $p <0.05$, *** $p < 0.001$ and ns by 1-way ANOVA.

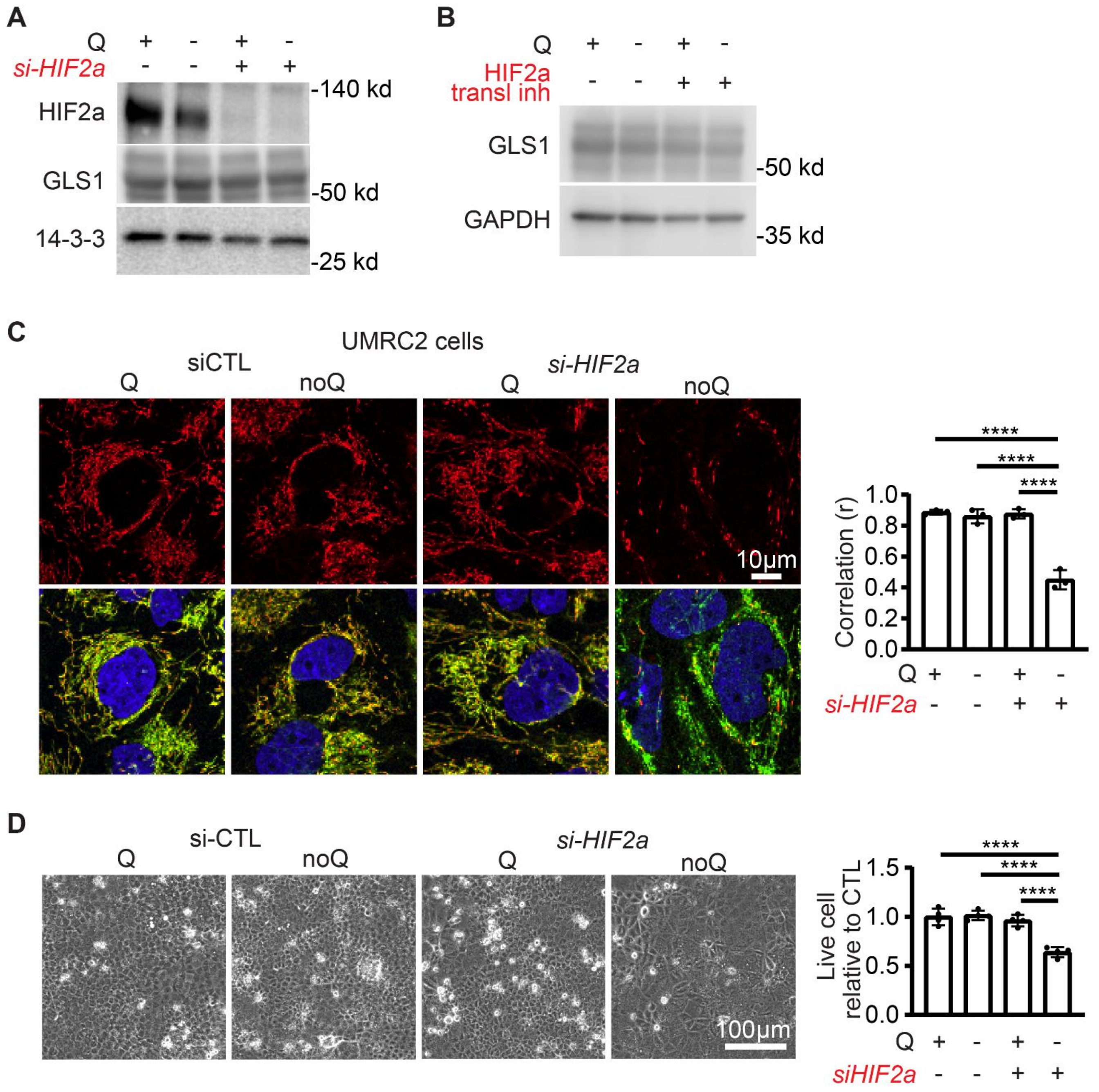

SFigure 7. GLS1 clustering is prevented in UMRC2 cells in a HIF2α-dependent manner.
A and **B**. Western blotting analysis showing the validation of *HIF2α* siRNA (A) and the effects of *si-HIF2α* siRNA (A) and its translational inhibitor (B) on the expression of GLS1 in UMRC2 cells. **C**. Resistance to noQ-induced GLS1 clustering in UMRC2 cells is reversed by *si-HIF2α*. **** $p < 0.0001$ by 1-way ANOVA. **D**. Resistance to noQ-induced cell death in UMRC2 cells is reversed by *siHIF2α*. **** $p < 0.0001$ by 1-way ANOVA.

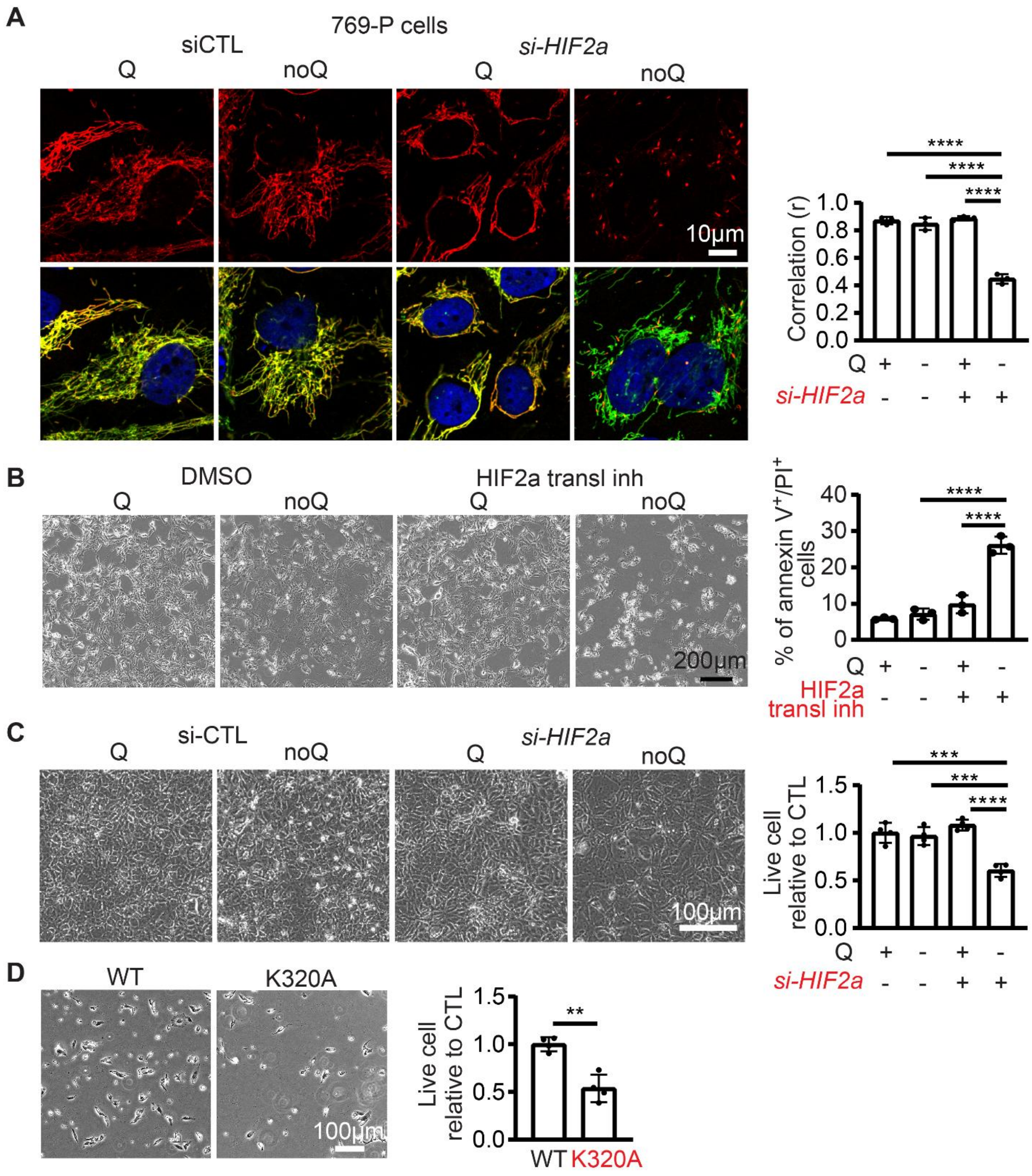

SFigure8. GLS1 clustering is prevented in 769-P cells in a HIF2α-dependent manner. A. Resistance to noQ-induced GLS1 clustering in 769-P cells is reversed by *si-HIF2α*. **** $p < 0.0001$ by 1-way ANOVA. **B**. Resistance to noQ-induced cell death in 769-P cells is reversed by treatment with an inhibitor of HIF2α translation. **** $p < 0.0001$ by 1-way ANOVA. **C**. Resistance to noQ-induced cell death in 769-P cells is reversed by *si-HIF2α*. **** $p < 0.0001$, *** $p < 0.001$ by 1-way ANOVA. **D**. Increased cell death in 769-P cells overexpressing the K320A mutant GLS1 compared to those overexpressing WT GLS. ** $p < 0.01$ by t-test.

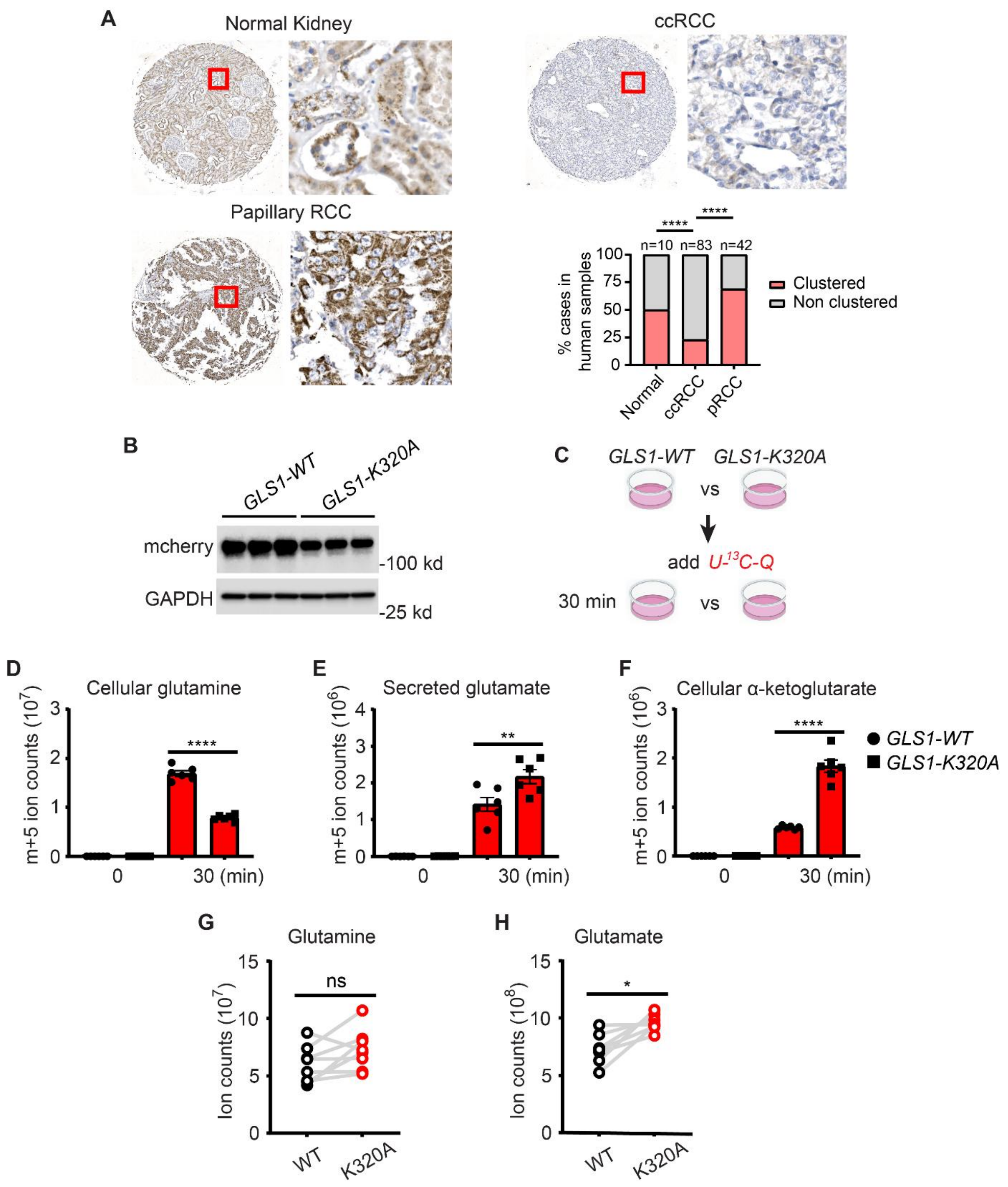

SFigure9. Enhanced enzymatic activity of GLS-K320A vs. GLS-WT in UMRC2 cells.

A. Reduced GLS1 clustering in human ccRCC samples compared to Papillary RCC. Images show the IHC of GLS1 in human clinical samples. Magnifications of the area in the block are shown on the right of each image. The number of the independent samples quantified in each group are shown on the top of the bar graph. **B**. Western blotting analysis showing the validation of the expression of GLS1-WT and GLS1-K320A in UMRC2 cells. **C**. Experimental scheme of U-^{13}C-Q tracing assay in UMRC2 cells. **D-F**. Quantifications of m+5 metabolites including glutamine (D), glutamate (E) and aKG (F) with conditions shown in C. ** $p < 0.01$ and **** $p < 0.0001$ by 1-way ANOVA. **G** and **H**. Quantifications of glutamine (G) and glutamate (H) in UMRC2 tumors. * $p < 0.05$ and ns by paired t-test.

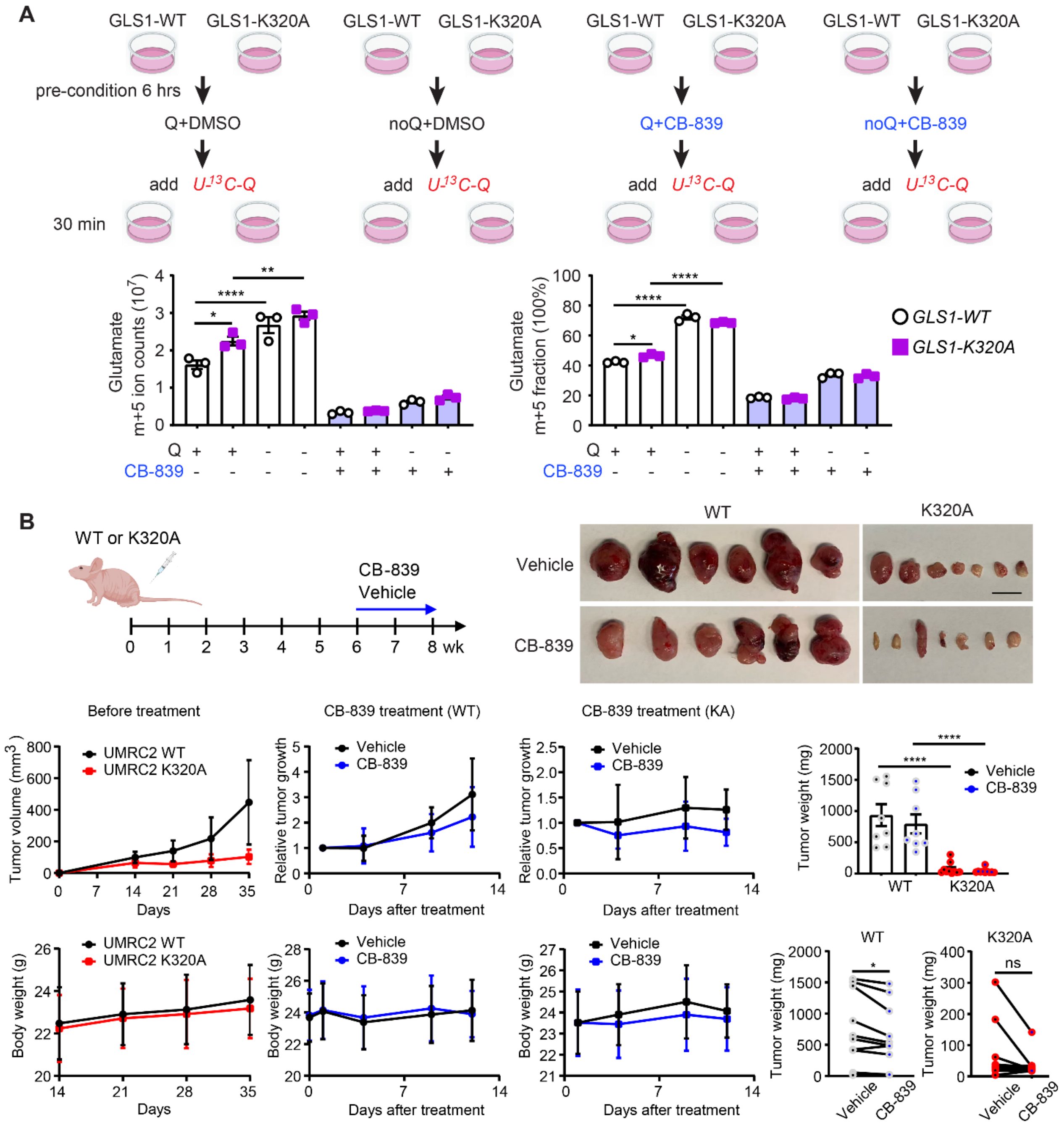

SFigure 10. The effect of GLS inhibition on UMRC2 tumors expressing GLS1-WT or GLS1-K320A.

A. U-^{13}C-Q tracing assay showing that CB-839, GLS1 inhibitor, significantly reduces the enzymatic activity of glutaminase in UMRC2 cells overexpressing GLS1-WT or GLS1-K320A. 2 μM CB-839 or DMSO was added at 6 hours before switching into the culture media with 2 mM U-^{13}C-Q. **B**. The effect of GLS1 inhibition on UMRC2 tumors. Mice were regrouped based on an assessment of tumor volume and body weight using a published tool RandoMice before the administration of CB-839. CB-839 treatment shows a trend to delay the growth of tumors expressing either GLS1-WT or GLS1-K320A (digital images and bar graphs). Overall, K320A tumor growth is remarkably inhibited compared with WT tumors (quantification of tumor weight at endpoint). The scale bar is 1 cm. **** $p < 0.0001$ by 1-way ANOVA, and * $p < 0.05$ or ns by paired t test.
