# Supplementary figures and images for: HIF2α inhibits glutaminase clustering in mitochondria to sustain growth of clear cell renal cell carcinoma
Source: JCI Insight. 2025 Oct 30;10(23):e182711. doi: 10.1172/jci.insight.182711 (PMC12890500; doi:10.1172/jci.insight.182711)

Fig1D

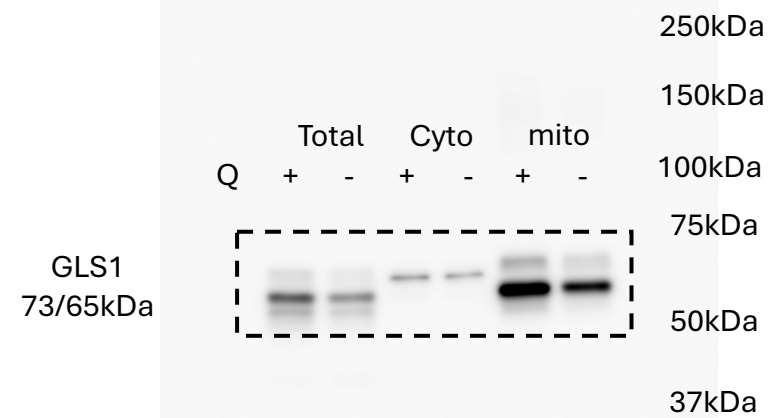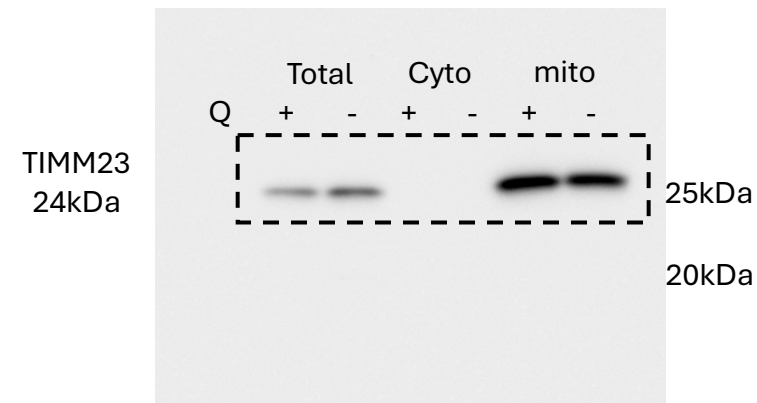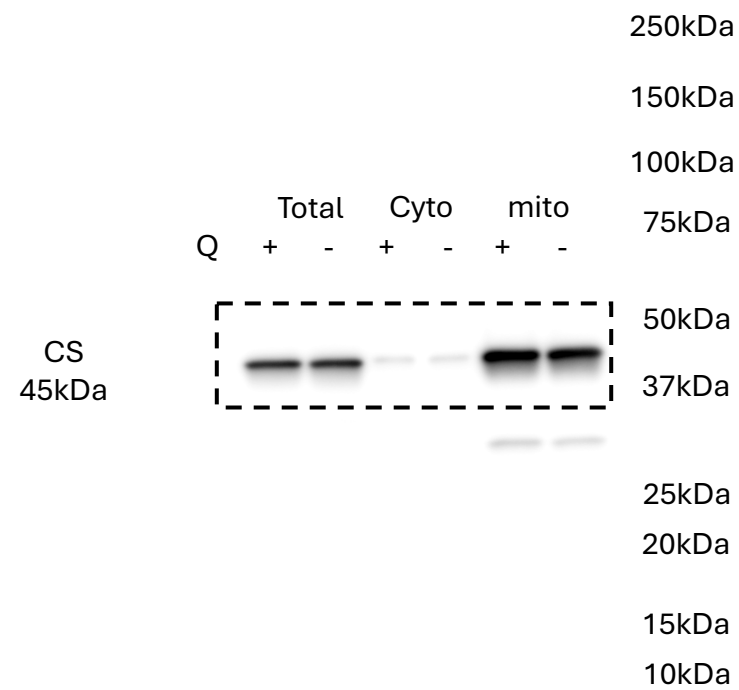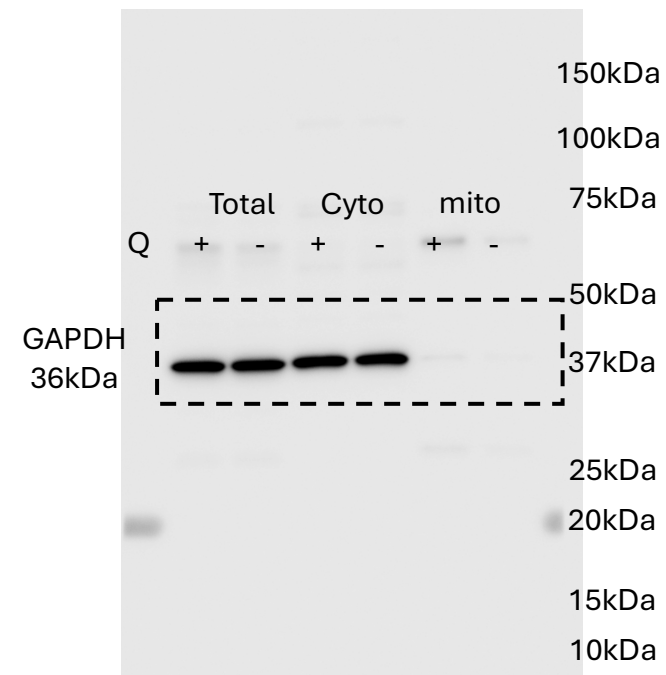

Fig4F

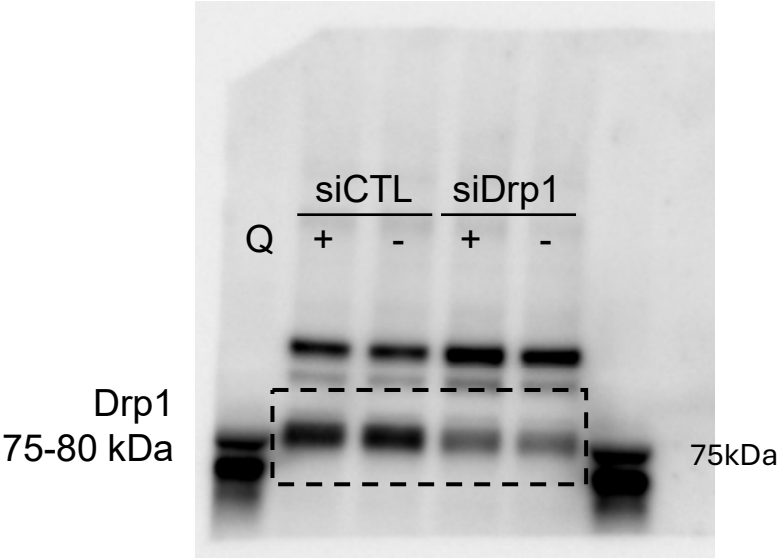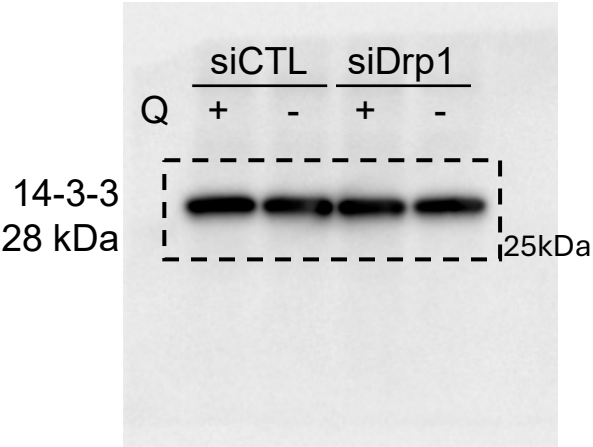

Fig5D

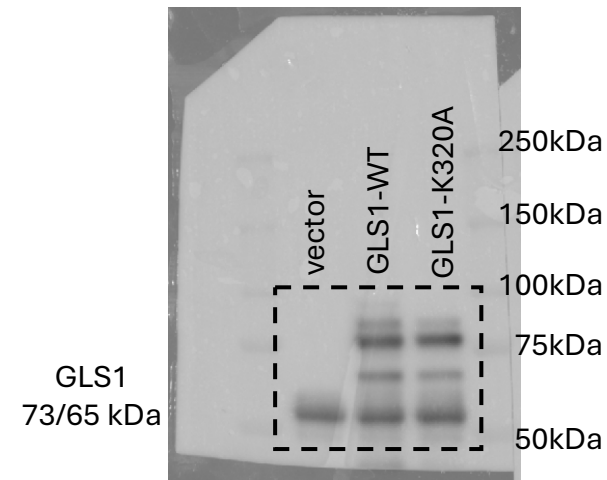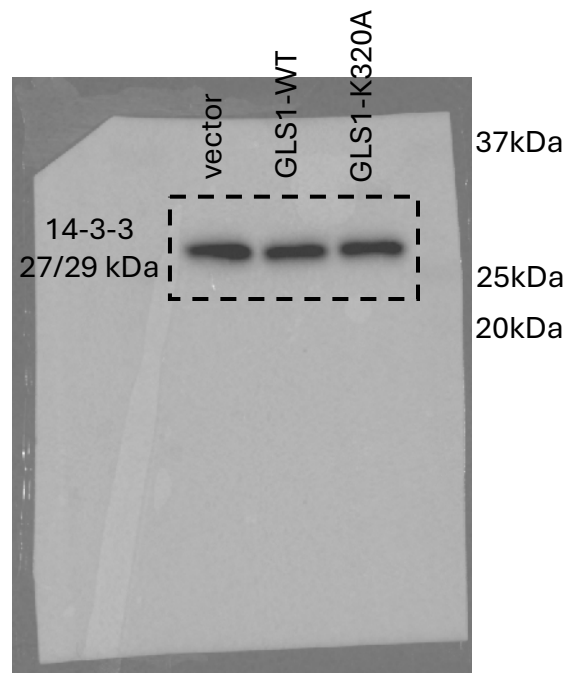

Fig6B

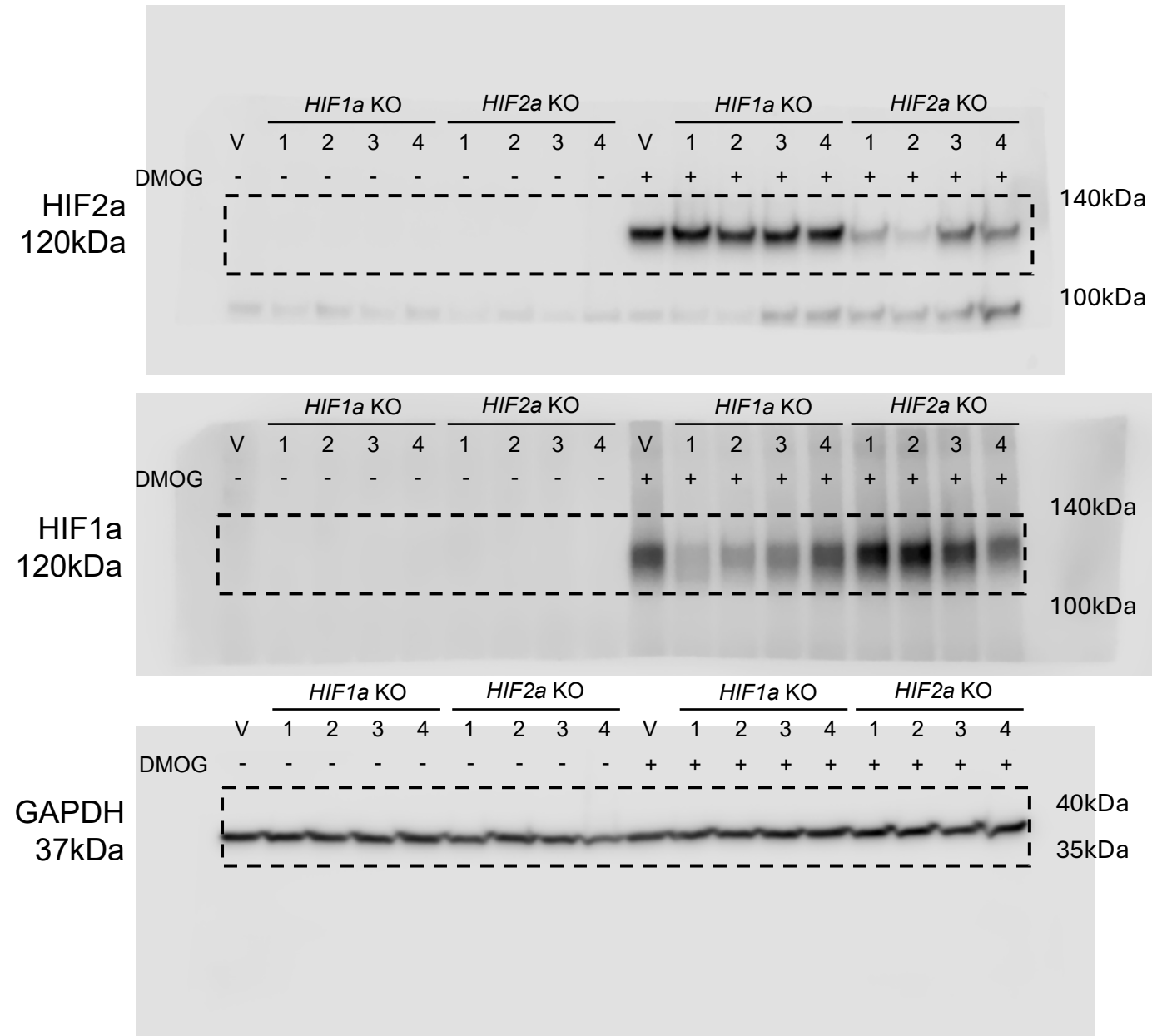

Fig7A

HIF2a  
120kDa

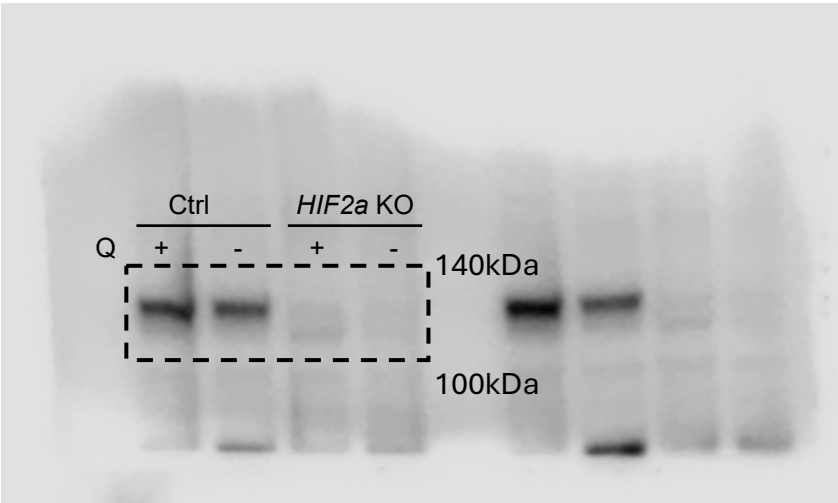

14-3-3  
28kDa

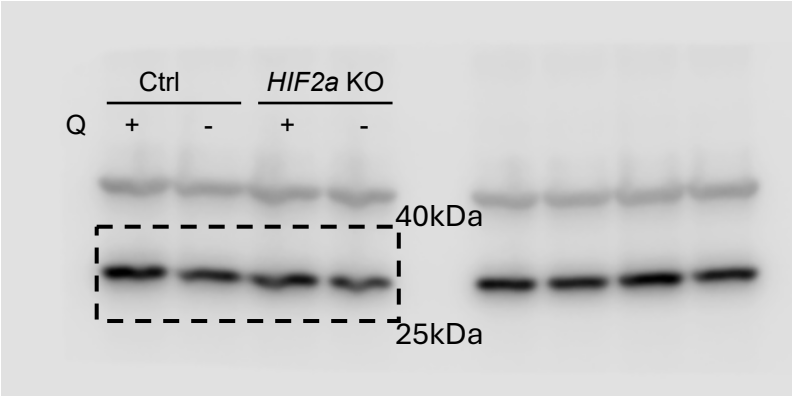

Fig7B

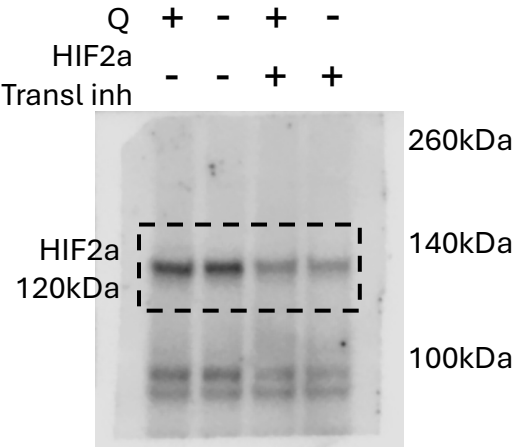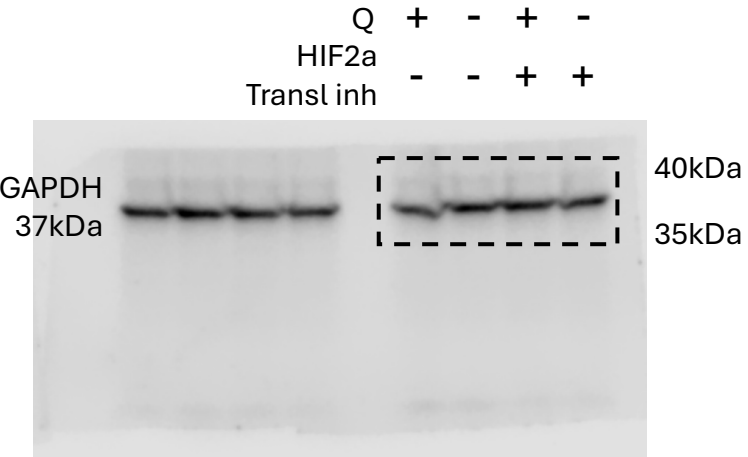

SFig6E

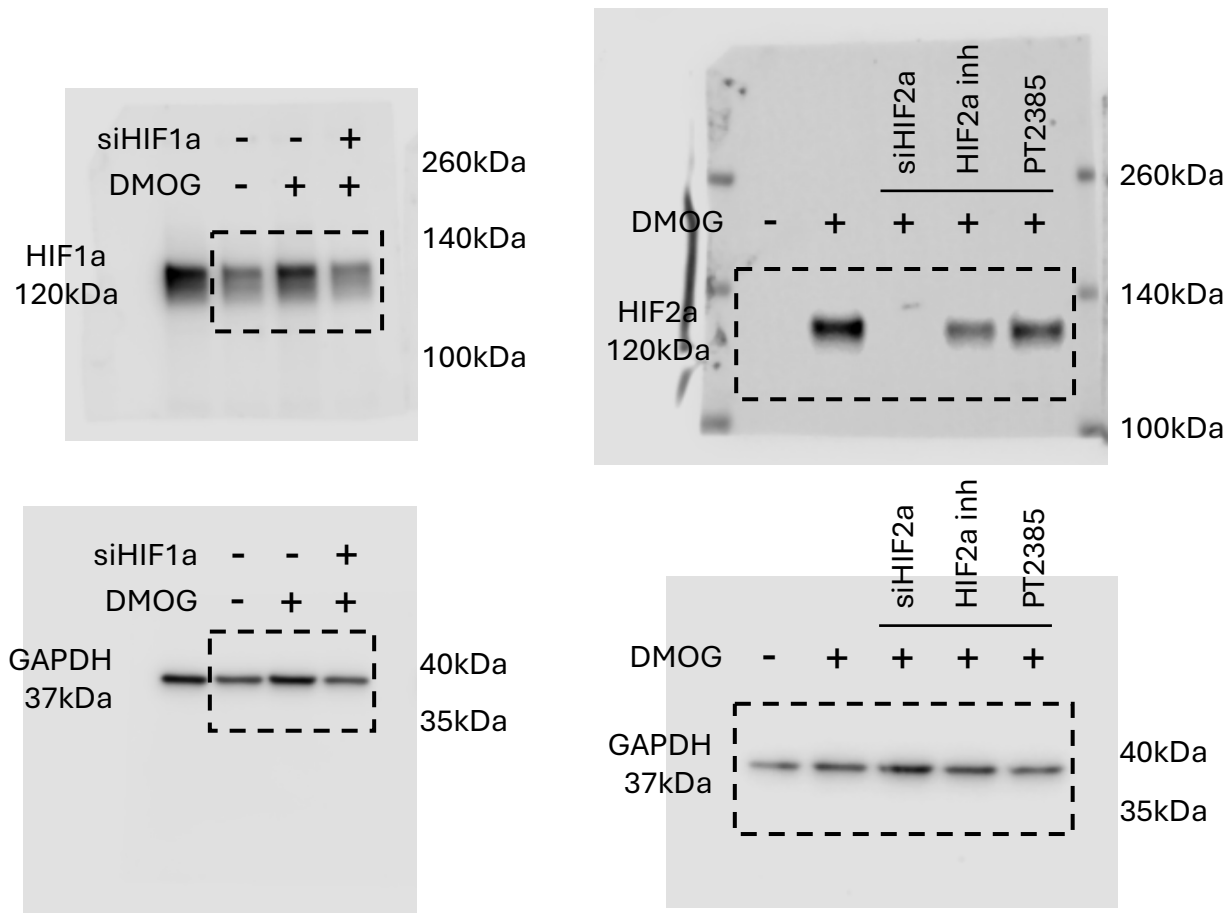

SFig6G

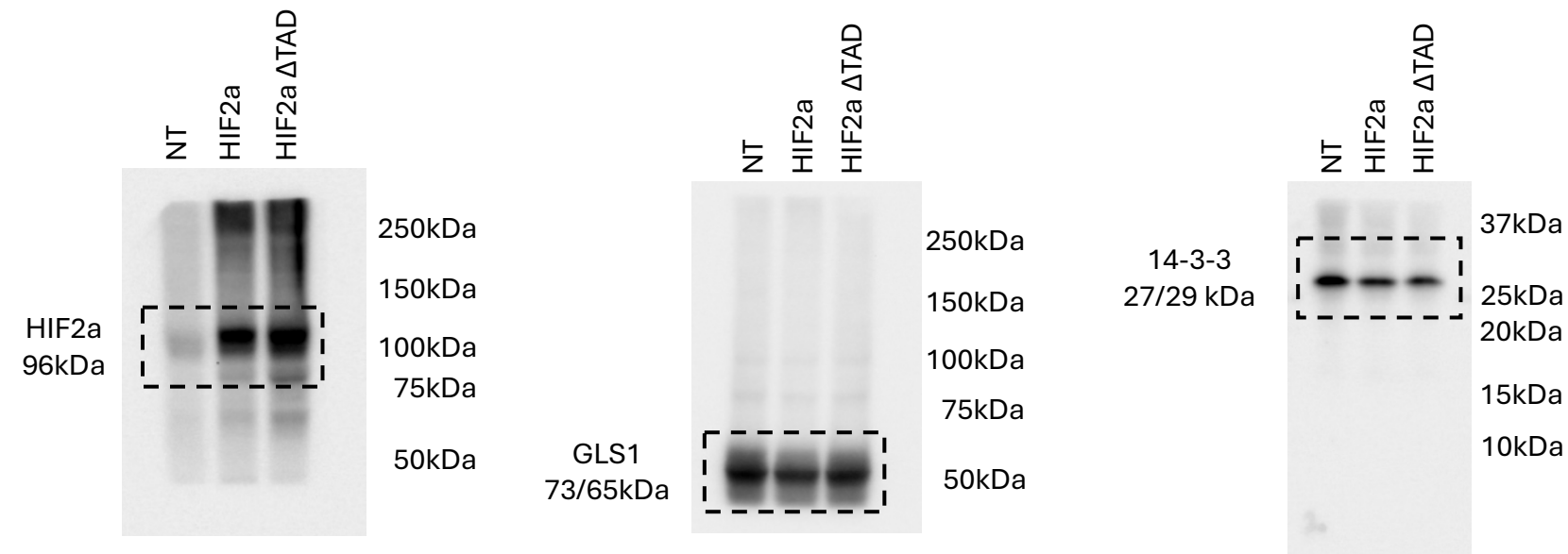

SFig7A

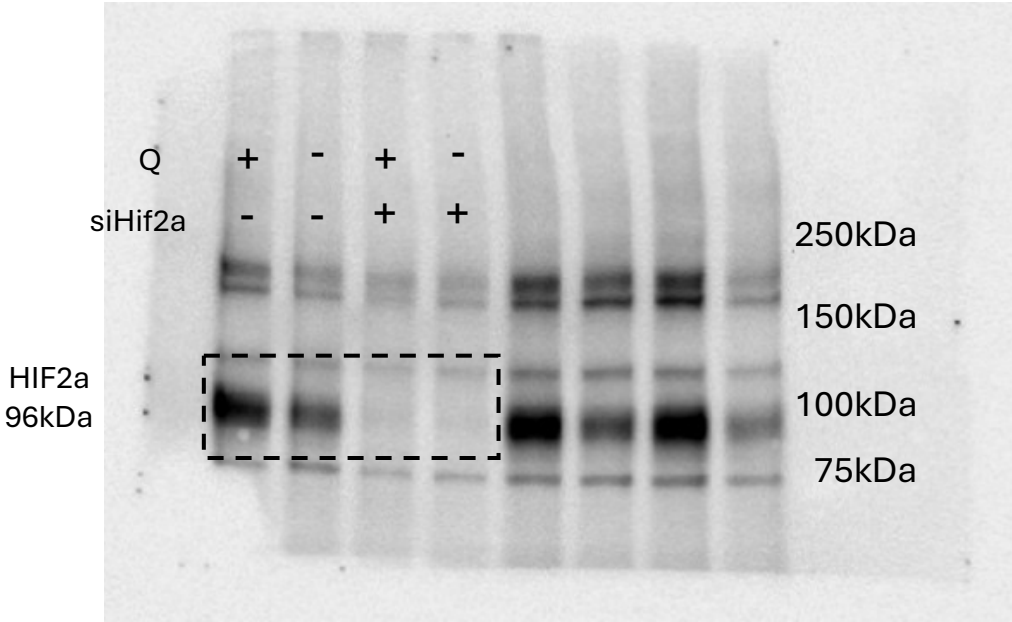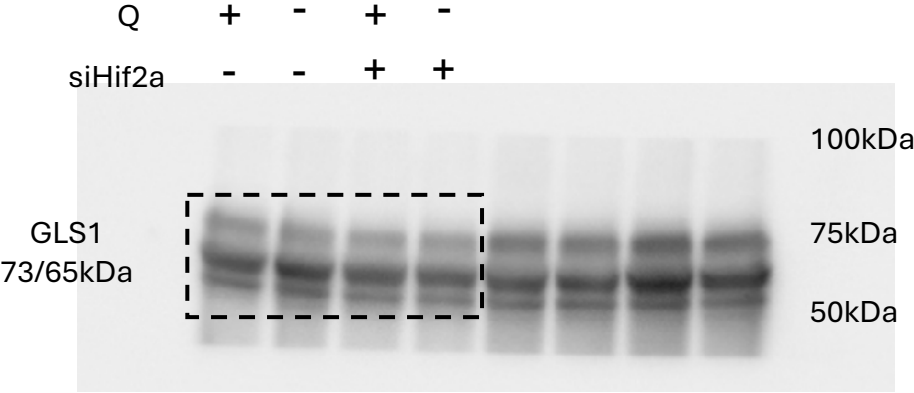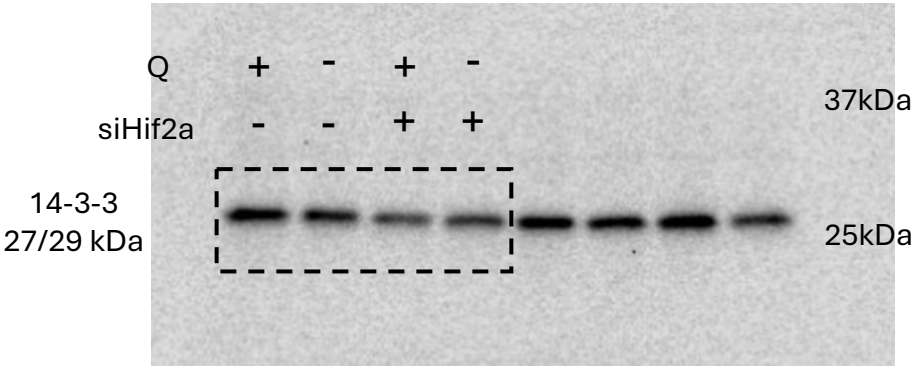

SFig7B

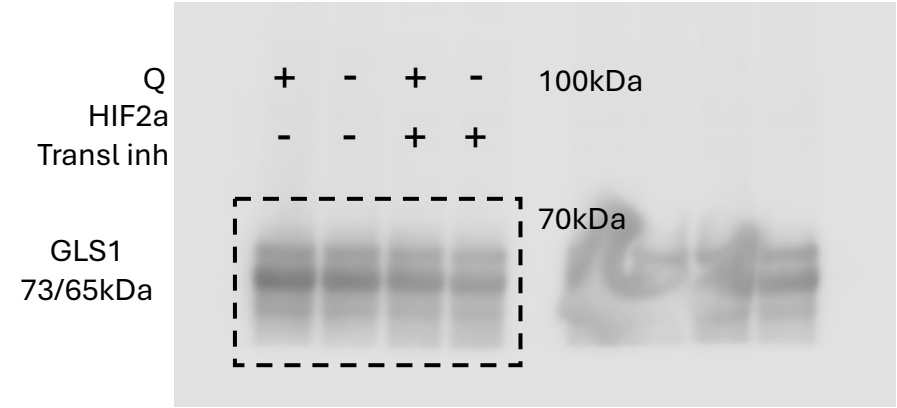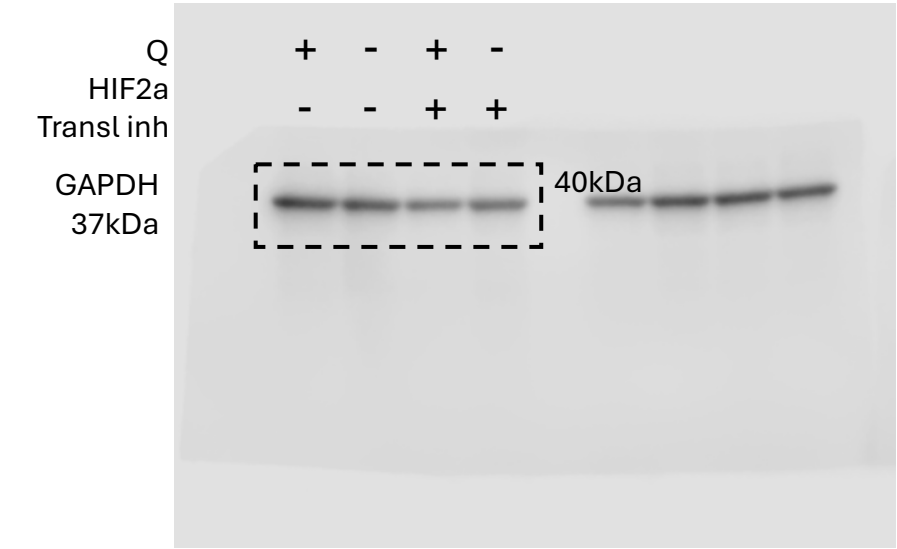

SFig9B

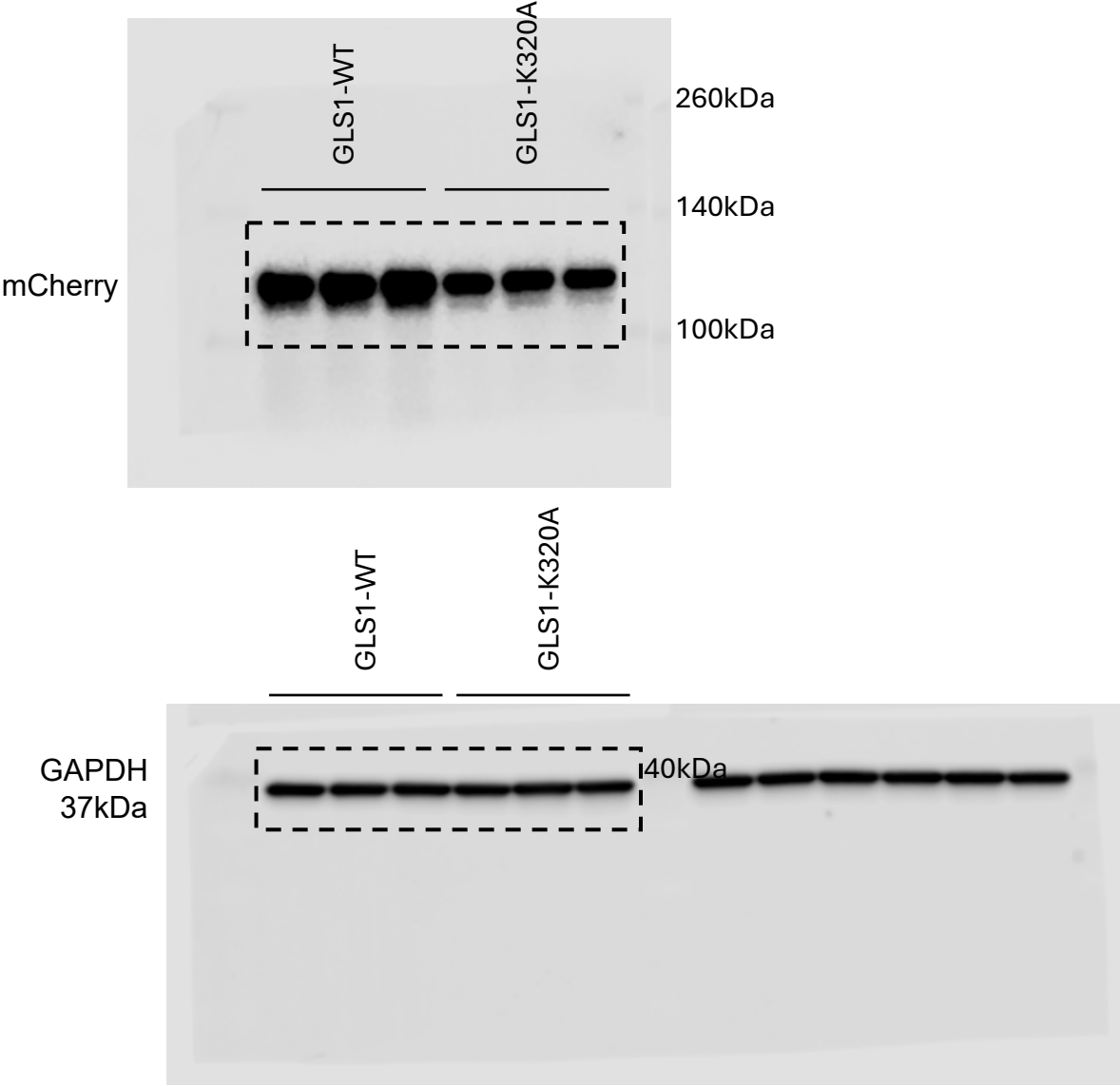

Supplement: Unedited blot and gel images [file jciinsight-10-182711-s127.pdf]
